# 25HC regulates the polarization of CD163^+^ macrophages in the immune microenvironment of triple-negative breast cancer through the interferon pathway

**DOI:** 10.3389/fimmu.2026.1812056

**Published:** 2026-05-28

**Authors:** Hongmei Zheng, Wenqin Huang, Panshi Zhang, Sudhanshu Bhushan, Xinhong Wu, Yalong Yang

**Affiliations:** 1 Department of Breast Center, Hubei Cancer Hospital, Tongji Medical College, Huazhong University of Science and Technology, Hubei Provincial Clinical Research Center for Breast Cancer, Wuhan Clinical Research Center for Breast Cancer, Wuhan, China; 2 Department of Thyroid and Breast Surgery, Tongji Hospital, Huazhong University of Science and Technology, Wuhan, China; 3 Department of Anatomy and Cell Biology, Unit of Reproductive Biology, Justus-Liebig-University Giessen, Giessen, Germany

**Keywords:** 25-hydroxycholesterol, cholesterol-25-hydroxylase, interferon regulatory factor 7, macrophage, polarization, triple-negative breast cancer

## Abstract

**Introduction:**

Triple-negative breast cancer (TNBC) is a highly aggressive subtype with limited therapeutic options. Reprogramming the tumor immune microenvironment (TIME) has emerged as a promising strategy for breast cancer treatment, and tumor-associated macrophages, the most abundant and heterogeneous immune cells in the solid TIME, play a pivotal role in this process. This study aimed to investigate the TNBC-specific TIME.

**Methods:**

Multiplex fluorescence immunohistochemistry and bioinformatic analyses were employed to map the immune landscape and predict potential regulatory mechanisms. These predictions were subsequently validated using *in vitro* cellular assays and *in vivo* mouse models. The unique lipid metabolic signature of TNBC was integrated with its distinct immune contexture to identify key regulators.

**Results:**

The oxysterol metabolite 25-hydroxycholesterol (25HC) was identified as a key regulator of macrophage polarization. Specifically, we discovered that an aberrantly activated interferon regulatory factor 7–cholesterol-25-hydroxylase–25HC axis in TNBC cells potently inhibits CD163^+^ macrophage polarization.

**Discussion:**

To our knowledge, this is the first study to delineate the link between dysregulated 25HC metabolism within the TIME and the control of macrophage polarization. These findings provide novel insights into the immunometabolic mechanisms underlying TNBC aggressiveness and reveal a potential therapeutic target for this devastating disease.

## 1 Introduction

Breast cancer remains the most prevalent malignancy and a leading cause of cancer-related mortality among women worldwide (1, 2). In China, it constitutes 12.2% of new cancer cases and 9.6% of cancer deaths (3). The advent of molecular classification has revolutionized breast cancer management, enabling precise diagnoses and targeted therapies that have significantly improved patient outcomes (4, 5). Despite these advances, triple-negative breast cancer (TNBC)-lacking expression of estrogen receptor (ER), progesterone receptor (PR), and human epidermal growth factor receptor 2 (HER2)-poses a major clinical challenge due to the absence of effective targeted therapies and its aggressive profile (6, 7). This unmet need underscores the importance of investigating alternative regulatory mechanisms, such as those governing cancer stem cells, apoptosis, and autophagy (8).

The tumor microenvironment (TME) is a complex ecosystem comprising not only tumor cells but also a myriad of stromal components, including fibroblasts, vascular endothelial cells, and infiltrating immune cells (9–11). These constituents play pivotal roles in tumor initiation, progression, and metastasis by facilitating tissue remodeling, angiogenesis, epithelial-mesenchymal transition, and modulating immune responses-either promoting immune escape or, paradoxically, driving excessive inflammation (10–14). Among these, tumor-associated macrophages (TAMs) constitute the most abundant immune cell population in the breast TME (15). TAMs are not a homogeneous entity but exhibit remarkable plasticity, encompassing a spectrum of phenotypes from classic pro-inflammatory (M1) to anti-inflammatory (M2) states, with numerous intermediate subtypes (15–17). While the M2 phenotype is traditionally viewed as pro-tumorigenic due to its role in immunosuppression and tissue repair (18), emerging evidence highlights that sustained pro-inflammatory (M1-like) responses can also contribute to tumorigenesis in certain contexts by fostering a mutagenic and growth-promoting environment (19). Therefore, the traditional classification of macrophages is being significantly challenged regarding its role in tumors, and numerous unresolved issues that require immediate attention persist. The tumor microenvironment of TNBC exhibits unique immunological features that distinguish it from other breast cancer subtypes. TNBC is characterized by higher levels of tumor-infiltrating lymphocytes (TILs) compared to hormone receptor-positive tumors, along with a prominent pro-inflammatory cytokine signature and elevated expression of immune checkpoint molecules (20). Paradoxically, despite this immune-rich microenvironment, TNBC remains the most aggressive subtype, suggesting that the nature of the immune response—rather than its mere abundance—may be qualitatively distinct. Understanding the specific immune-regulatory mechanisms that shape the TNBC microenvironment is therefore of critical importance.

The oxysterol metabolite 25-hydroxycholesterol (25HC), an endogenous product of cholesterol metabolism, is increasingly recognized as a critical immunomodulator. Beyond its well-characterized antiviral activities, 25HC exerts profound effects on both innate and adaptive immunity (21–23). Notably, 25HC can influence macrophage function and polarization in a context-dependent manner (24); however, its precise role within the cancer immune microenvironment, particularly in TNBC, remains poorly defined. In the context of cancer, 25HC has been studied in several tumor types with sometimes opposing effects: in colorectal cancer, 25HC enhances the immunosuppressive function of myeloid-derived suppressor cells (MDSCs) (25); in melanoma, lymphatic endothelial cell-derived 25HC promotes anti-tumor immunity by reprogramming macrophages toward a pro-inflammatory phenotype (26); and in breast cancer, 25HC has been implicated in chemoresistance through oxysterol-Pgp axis activation (27). However, the precise role of 25HC within the TNBC immune microenvironment remains poorly defined, and its potential function in regulating macrophage polarization in TNBC has not been previously reported.

Given the peculiarities of the TNBC immune landscape and its association with aberrant lipid metabolism, we hypothesized that 25HC might serve as a key metabolic mediator shaping macrophage polarization in TNBC. In this study, we first comprehensively profiled the immune microenvironment in human breast cancer specimens using multiplex fluorescence immunohistochemistry. Through integrated bioinformatic analysis and experimental validation, we identified the interferon regulatory factor 7 (IRF7)-cholesterol-25-hydroxylase (CH25H)-25HC axis as a novel pathway dysregulated in TNBC that critically regulates macrophage polarization and tumor progression.

## 2 Methods

### 2.1 Mouse

Female BALB/c nude and C57BL/6J wild-type mice (6–8 weeks old, 25 ± 1.5 g) were purchased from Gempharmatech Co., Ltd. (Jiangsu, China). Mice were housed under standard specific pathogen-free conditions (22 °C, 12-hour light/dark cycle) with free access to food and water. All animal experimental procedures were approved by the Laboratory Animal Welfare and Ethics Committee of Zhongnan Hospital, Wuhan University (*License No. 20200702*) and conducted in accordance with the NIH Guide for the Care and Use of Laboratory Animals.

### 2.2 Cell culture and lentiviral transfection

The human breast cancer cell lines MCF-7, MDA-MB-231, MDA-MB-468, AU-565, the murine TNBC cell line 4T1, and the human monocytic cell line THP-1 were obtained from the American Type Culture Collection. The human normal mammary epithelial cell line MCF-10A was cultured in specialized mammary epithelial cell growth medium (CM-0525, Procell, China). All other cell lines were maintained in Dulbecco’s Modified Eagle Medium (DMEM; Gibco, USA) supplemented with 10% fetal bovine serum (FBS; Cellbox, #AUS-01S-02, China) and 1% Penicillin-Streptomycin Solution (*Procell, China*) at 37 °C in a 5% CO_2_ humidified incubator.

For lentiviral transduction, 2×10^5^ MDA-MB-231 or 4T1 cells were seeded per well in 6-well plates. After 12 h, cells were transfected with lentiviral particles (PGMLV-hU6-MCS-CMV-ZsGreen1-PGK-Puro-WPRE) at the appropriate multiplicity of infection (MOI) in the presence of 5 μg/mL polybrene. The medium was replaced 12 hours post-transduction. Selection was initiated 72 hours later using 1 μg/mL puromycin, and fresh selection medium was replaced every 2–3 days. Stable polyclonal populations were established after four weeks of selection and validated by fluorescence microscopy and functional assays.

### 2.3 *In vitro* macrophage polarization model

THP-1 monocytes were differentiated into macrophages (regarded as M0) by treatment with 100 ng/mL phorbol 12-myristate 13-acetate (PMA; HY-18739, MCE, USA) for 48 hours (28). To induce polarization, M0 macrophages were then treated for an additional 48 hours with either: 1) 100 ng/mL lipopolysaccharide (LPS; L2630, Merck, USA) + 20 ng/mL interferon-γ (IFN-γ; *SRP3058, Merck, USA*) to generate M1 macrophages, or 2) 20 ng/mL interleukin-4 (IL-4; H7291, Merck, USA) + 20 ng/mL interleukin-13 (IL-13; I1771, Merck, USA) to generate M2 subtype (29).

### 2.4 Flow-based multi-indicator enzyme immunoassay

Cell supernatants were collected and centrifuged at the highest speed for 10 min to remove cell debris. Antibody molecules for different substances to be tested were covalently crosslinked to specifically coded microspheres, with each coded microsphere corresponding to a specific assay (ABplex Human 21-Plex Customs Panel, RK04350, Abclonal, China). A concentration gradient of standards for each substance to be tested was constructed, the samples to be tested and the microspheres labeled with each substance were incorporated, washed by magnetic field adsorption, and coupled with fluorescein, and the data were collected and evaluated using an Abplex-100 flow-through analyzer.

### 2.5 Real-time quantitative polymerase chain reaction

Total RNA was extracted with the RNeasy Mini Kit (Qiagen, Germany) according to the manufacturer’s instructions. RNA concentrations were measured using a NanoDrop 2000/2000c spectrophotometer (Thermo Fisher Scientific, USA). Reverse transcription was performed using ABScript III RT Master Mix for qPCR with gDNA Remover (*Abclonal, RK20429, China*). Quantitative real-time PCR (qRT-PCR) was carried out with 2× Universal SYBR Green Fast qPCR Mix (Abclonal, RK21203, China) on an iCycler iQ^®^ System (Bio-Rad, Munich, Germany), and data were analyzed using Bio-Rad CFX Manager 3.1 software (Bio-Rad, USA). Gene expression levels were normalized to the housekeeping gene *GAPDH*. All CT values represent the average of triplicate measurements. The ΔCT was calculated as CT (target gene) – CT (GAPDH). Untreated samples served as the control group, and the ΔΔCT for treated samples was determined as ΔΔCT = ΔCT (treated) – ΔCT (control). Relative fold changes were calculated using the formula 2^(-ΔΔCT). Final gene expression levels are presented relative to the control group, which was set to a value of 1. The primer sequences are listed in **Supplementary Table 1**. The cycle for qRT-PCR was as follows: initial melting at 95 °C, 10 min; 40 cycles including 95 °C, 20 s melting, 58 °C, 30 s for annealing (63 °C for IRF7), and 72 °C, 30 s for extension; 50 °C, 10 s for melting point.

### 2.6 Immunohistochemistry

Breast cancer tissue was collected from Zhongnan Hospital and approved by the Ethics Committee. The total number of samples was 107 (HER2 positive n=15; Luminal A n=19; Luminal B n=17; TNBC n=24; non-cancer n=32). The slides were deparaffinized and rehydrated. After antigen retrieval with boiling sodium citrate buffer (10 mM, pH6.0), the slide was incubated with 3% H_2_O_2_ and blocked with 10% goat serum, followed by incubation with IRF7 antibody (ab115352, Abcam, USA) (4 °C, overnight) and goat anti-rabbit HRP secondary antibody (#S0001, Affinity, China; 30 min, RT). Subsequently, the slides were stained with 3,3′-diaminobenzidine (DAB) and counterstained with hematoxylin. IRF7 expression was quantitatively evaluated by enumerating the percentage of positive cells using ImageJ software 1.53e.

### 2.7 Enzyme-linked immunosorbent assay

Following treatment, cell culture supernatants were collected and evaluated using human-25HC ELISA kits (EY3671, Yiyan, China) according to the manufacturer’s guidelines.

### 2.8 Immunofluorescence

Cells were grouped according to the experimental purpose and inoculated into a six-well plate with a glass plate at the bottom. After the different cell groups were treated accordingly, the supernatant was discarded and washed thrice with PBS, and 4% paraformaldehyde was incorporated for cell fixation for 10 min, followed by 3×5 min washes with PBS on a shaker. The cells were permeabilized by incorporating 0.05% Triton-100 for 30 min and blocked for 30 min with goat serum to bind non-specific binding. After washing, specific primary antibodies (CD11, #DF7585; CD68, #DF7518; CD163, #DF8235, AffinityBiotech, China) diluted in 1% BSA working solution were incorporated at 4 °C overnight incubation. The sections were then washed 3×5 min, and a secondary antibody [Goat Anti-Rabbit IgG (H+L) Fluor594-conjugated, #S0006, Affinity Biotech, China] solution was incorporated and incubated for 1 h in the dark. Nuclei were stained with DAPI (Affinity Biotech, China) for 1 min, and slides were mounted with an anti-fluorescence quenching medium. Images were captured using a fluorescence microscope for analysis.

### 2.9 Mouse tumor model

**Figure 1:**
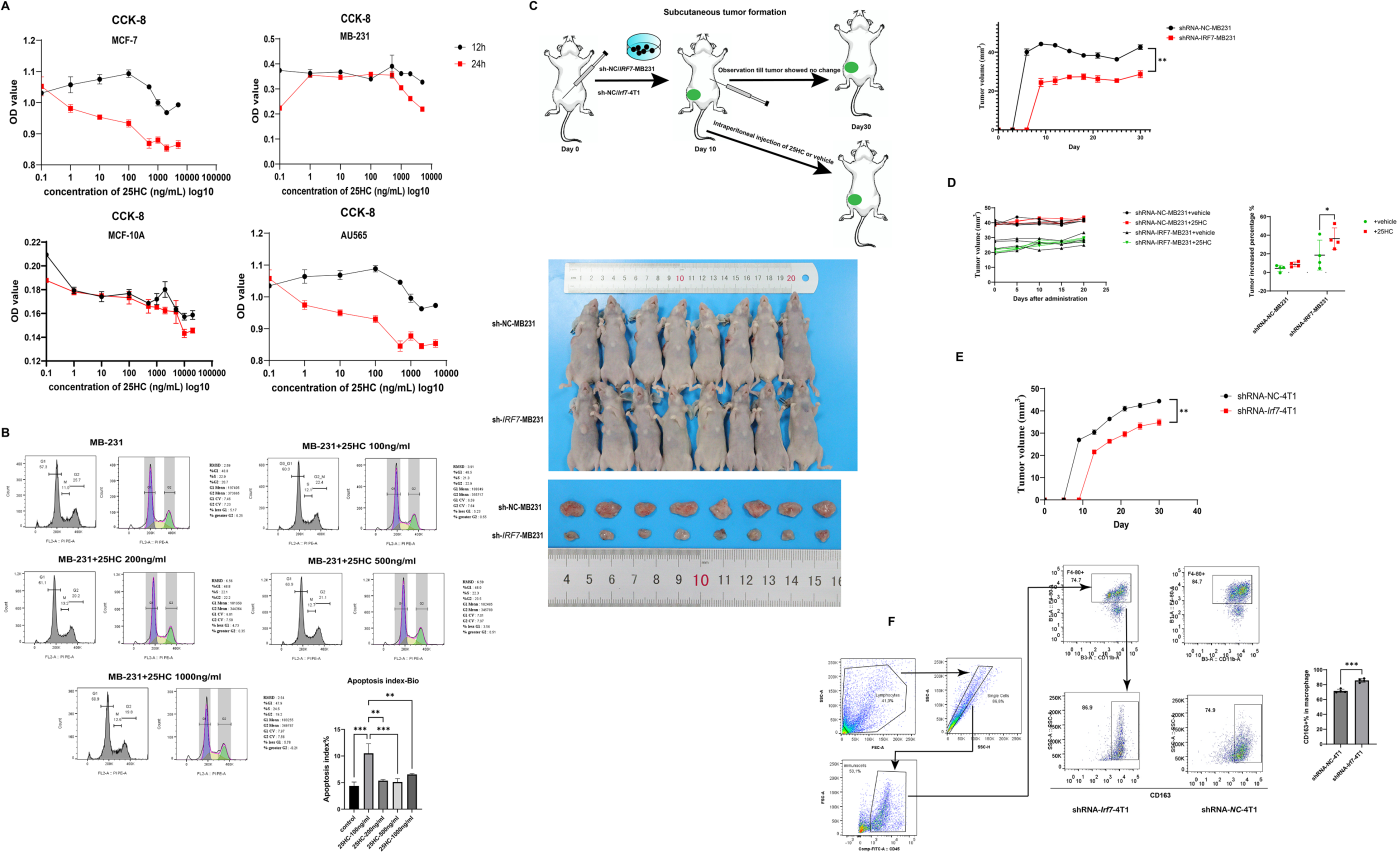
Role of *IRF7/CH25H/25HC* on the proliferative capacity of breast cancer. **(A)** CCK-8 assay is used to measure the proliferative capacity of diverse cell lines with diverse concentrations of 25HC. N = 3. **(B)** FC assay for the effect of 25HC on the TNBC cell cycle. N = 4. **(C)** Inhibition of *IRF7* expression status in TNBC cells inhibits tumorigenicity *in vivo*. N = 8. **(D)** Intervention with 25HC significantly reverses the slowing of tumor growth brought about by reduced *IRF7*. N = 4. **(E)** Inhibition of Irf7 expression status in 4T1 cells inhibits tumorigenicity *in vivo*. N = 4. **(F)** CD163+ percentage in TME between shRNA-*Irf7*-4T1 and shRNA-NC-4T1. N = 4. The data are evaluated with the 2-way and one-way ANOVA with the Tukey test or 2-tailed unpaired t-test. *p<0.05; **p<0.01; ***p<0.005.

We used two mouse models due to the deficiency of the immune system in nude mice. MB-231 cells were injected into nude mice, and 4T1 cells were injected into wild-type (WT) mice. A total of 2×10^6^ shRNA-*IRF7*-MB-231 or sh-NC-MB-231 cells (shRNA-Irf7-4T1 or shRNA-NC-4T1 cells for WT mice) were collected in PBS and injected subcutaneously into the interstitial space of the right hind limbs. Mice were checked daily, and tumor length and width were measured using digital calipers every 3 days starting from day 10 post-injection. Tumor volume was calculated using the formula: volume = (length × width²)/2. The endpoint criteria were: (1) tumor volume exceeding 1500 mm³, (2) tumor ulceration or necrosis affecting >25% of tumor surface, (3) body weight loss exceeding 20%, or (4) signs of severe distress (hunched posture, ruffled fur, lethargy). For the xenograft model (**Figure 1C**), shRNA-IRF7-MB-231 group (N = 8) and sh-NC-MB-231 group (N = 8) were injected. For the 25HC intervention study (**Figure 1D**), mice in the treatment groups were intraperitoneally injected with 25HC (10 mg/kg in 5% DMSO + 95% PBS) or vehicle control daily from day 10 to day 20, with N = 4 per group. For the syngeneic 4T1 model (**Figure 1E**), shRNA-Irf7-4T1 group (N = 4) and shRNA-NC-4T1 group (N = 4) were used. All animals were euthanized at day 20 post-injection or when endpoint criteria were met, whichever came first. At the end of the study period, tumors were collected, measured, and fixed for IHC and flow cytometry.

### 2.10 Flow cytometry

FC analysis was conducted as described previously as follows. Single-cell macrophage suspensions were obtained by digestion with 0.05% trypsin-EDTA. After centrifugation, cell pellets were resuspended in FC buffer (1% BSA [w/v in DPBS] + 10 mM EDTA), blocked with an Fc-blocking antibody (anti-CD32/16, *BD Pharmingen, USA*) for 10 min at 4 °C and then incubated with macrophage-specific antibodies (all from Biolegend, USA, PerCP/Cyanine5.5, anti-human CD11b Antibody, Cat#393106; FITC anti-human CD11c Antibody, Cat#337214; APC anti-mouse CD68 Antibody, Cat#137008; APC anti-human CD163 Antibody, Cat#326510). After staining, analysis was conducted using a CytoFLEX LX (*Beckman Coulter, USA*). Data were evaluated using FlowJo software v10.5 (*Tree Star, USA*).

### 2.11 Western blotting

Total cell lysates were obtained using RIPA buffer (P0013B, Byotime, China) containing protease inhibitor PMSF (Selleck, USA). Proteins were separated by SDS-PAGE and transferred to PVDF membranes (Millipore, USA). The bands were visualized using the ECL chemiluminescence kit (PMK003, Pumoke, China). IRF7 Rabbit mAb (A22742) and GAPDH Rabbit mAb (A19056) were purchased from Abclonal, China.

### 2.12 Multiplexed immunofluorescence

Multiplex immunofluorescence was conducted using the PerkinElmer Opal 7-Color Manual IHC kit (NEL811001KT; PerkinElmer, Waltham, MA, USA). The tissue chip was acquired as 4 µm thick with 1 µm diameter for each sample. After deparaffinization, the chip sections were rehydrated, the antigen was restored in Tris-EDTA buffer (pH 9.0) using a microwave, and 3% H_2_O_2_ was used to inactivate endogenous peroxidase. The following steps of multiplexed IHC were conducted consecutively for each marker: blocking non-specific markers with goat serum (G9023, Sigma, USA), followed by incubation with primary antibody for 1 h, detection using Opal Polymer HRP Ms/Rb secondary antibody (PerkinElmer, Waltham, MA, USA), and visualization using Opal tyramide signal amplification (TSA)-fluorescein (diluted with Amplification Dilution), after which the sections were placed in 1×AR6 Buffer and heated using a microwave. The primary antibodies and corresponding TSA used for each protein were as follows: Opal 520, anti-CD3 (60181-1-Ig); Opal 540, anti-CD68 (66231-2-Ig); Opal 570, anti-CD163 (16646-1-AP); Opal 620, anti-CD19 (66298-1-Ig); Opal 650, anti-CD40 (66965-1-Ig); and Opal 690, anti-TLR4 (22048; Abcam, USA). Primary antibodies were purchased from Proteintech (Rosemont, NY, USA) unless otherwise mentioned.

### 2.13 Multispectral image scanning and quantitative analysis

After staining with six-opal TSA-fluorescein and nuclear staining with DAPI, the spectral information of the tissue chip was captured and split using the Vectra 3.0 Automated Quantitative Pathology Imaging System (PerkinElmer, USA). The stained sections were scanned at 20x (multiplexed IHC and hematoxylin/eosin staining). The image files created by Vectra were then evaluated using Inform 2.1 image analysis software (PerkinElmer, USA). Owing to the heterogeneity during tissue preparation and staining of 151 dots in the chip, it was inaccurate to set up a single spectral library for batch analysis of all samples; instead, individual analysis was performed. As depicted in the analysis flowchart (**Supplementary Figure 1**), each fluorescence intensity was first adjusted for proper visual performance. The tumor/gland or stromal segment was selected based on classical histological characteristics, followed by automatic segment division. General and total immune profiles were also investigated. Nuclear and cytoplasmic analysis values were determined based on nuclear size, nuclear-splitting sensitivity, membrane thickness, and other parameters to precisely select single cells. At least five typically positive cells for each phenotype were selected, and the total number of sample dots was used for the cell phenotype map. The positive cell proportion was scored according to the fluorescence intensity in the double-positive analysis, exporting the percentage data of single-positive CD3, CD19, and CD68 and double-positive CD68-CD163, CD68-TLR4, and CD68-CD40 for further analysis.

### 2.14 Bioinformatic analysis

We downloaded RNA-seq data (FPKM normalized) and corresponding clinical information for breast cancer from The Cancer Genome Atlas (TCGA, https://portal.gdc.cancer.gov). Data filtering criteria were: (1) exclusion of metastatic samples (samples with M1 stage, N = 23); (2) exclusion of samples with incomplete follow-up information (survival time <30 days or missing survival status, N = 41); (3) exclusion of samples with ambiguous molecular subtype classification (N = 18). After filtering, 1,109 primary breast cancer samples and 113 corresponding paired non-cancerous tissue samples were included for analysis. To evaluate immune cell infiltration, the CIBERSORT algorithm (v1.04) was applied with the LM22 signature matrix, which defines 22 human immune cell subtypes. The algorithm was run with the following parameters: number of permutations = 1,000, quantile normalization disabled, and absolute mode enabled to obtain absolute infiltration scores. Samples with CIBERSORT output p-value >0.05 were excluded from downstream immune infiltration analysis to ensure reliability. The Wilcoxon rank-sum test was used to compare immune cell infiltration between groups. Survival analysis was performed using the Kaplan-Meier method with log-rank test, and multivariate Cox proportional hazards regression analysis was conducted to identify independent prognostic factors, with statistical significance set at p < 0.05.

We also downloaded the GSE42568 dataset (raw CEL and GPL files) from the GEO database, which is based on the GPL570 Platform [(HG-U133_Plus_2) Affymetrix Human Genome U133 Plus 2.0 Array] and contains 104 primary breast cancer and 17 normal breast biopsy gene chips. After quality control, a total of 94 cancer and 14 normal breast samples were obtained, based on which we identified the DEGs in breast cancer compared with normal samples using R package “limma” with log2Fold Change >1 and an adjusted p-value <0.05. The IRGs were obtained from the ImmPort database (https://www.immport.org/home). The Venn diagram was constructed using an online tool (http://bioinformatics.psb.ugent.be/webtools/Venn/) to identify the overlapping genes between DEGs and IRGs. We employed the STRING (https://www.string-db.org) tool to construct a PPI network of overlapping genes with a Pearson correlation coefficient of ≥ 0.4 and reproduced the network using Cytoscape. Subsequently, the plug-in MCODE of Cytoscape was used to detect the densely connected regions (i.e., modules) of networks, the criteria of which were a degree cut-off = 2, MCODE scores ≥ 4, Max. Depth = 100, k-score = 2, and node score cut-off =0.2. Enrichment analysis was conducted using the MetaScape software (http://metascape.org).

### 2.15 Patients

Patients with breast cancer who underwent surgery provided written informed consent for themselves after receiving comprehensive information about the study purpose, procedures, and data usage. The MI/IHC in this study was conducted using a tissue chip produced from the breast tissues of 151 patients from Zhongnan Hospital, including benign and malignant cases. Patients with breast cancer who underwent surgery provided informed consent. This study included 18 benign cases, 10 para-cancer samples, and 123 malignant cases, which included 37 Luminal A, 32 Luminal B, 35 HER2^+^, and 19 TNBC subtypes, according to the expression standard of the National Comprehensive Cancer Network guidelines for 2021 (30) (**Supplementary Table 2**). Zhongnan Hospital Ethical Committee approved the protocol of this study, and all data obtained from patients strictly complied with the relevant ethical requirements (*ZN2023049*).

### 2.16 Statistical analysis

The data were evaluated using a two-way and one-way ANOVA with the Tukey test or a 2-tailed unpaired t-test to express the differences in immune cell densities among diverse types or segments. GraphPad Prism 9 software (GraphPad, CA, USA) was used for the statistical analyses. Statistical significance was set at p < 0.05.

## 3 Results

### 3.1 The immune microenvironment of TNBC is characterized by reduced CD163^+^ macrophage infiltration

**Figure 2:**
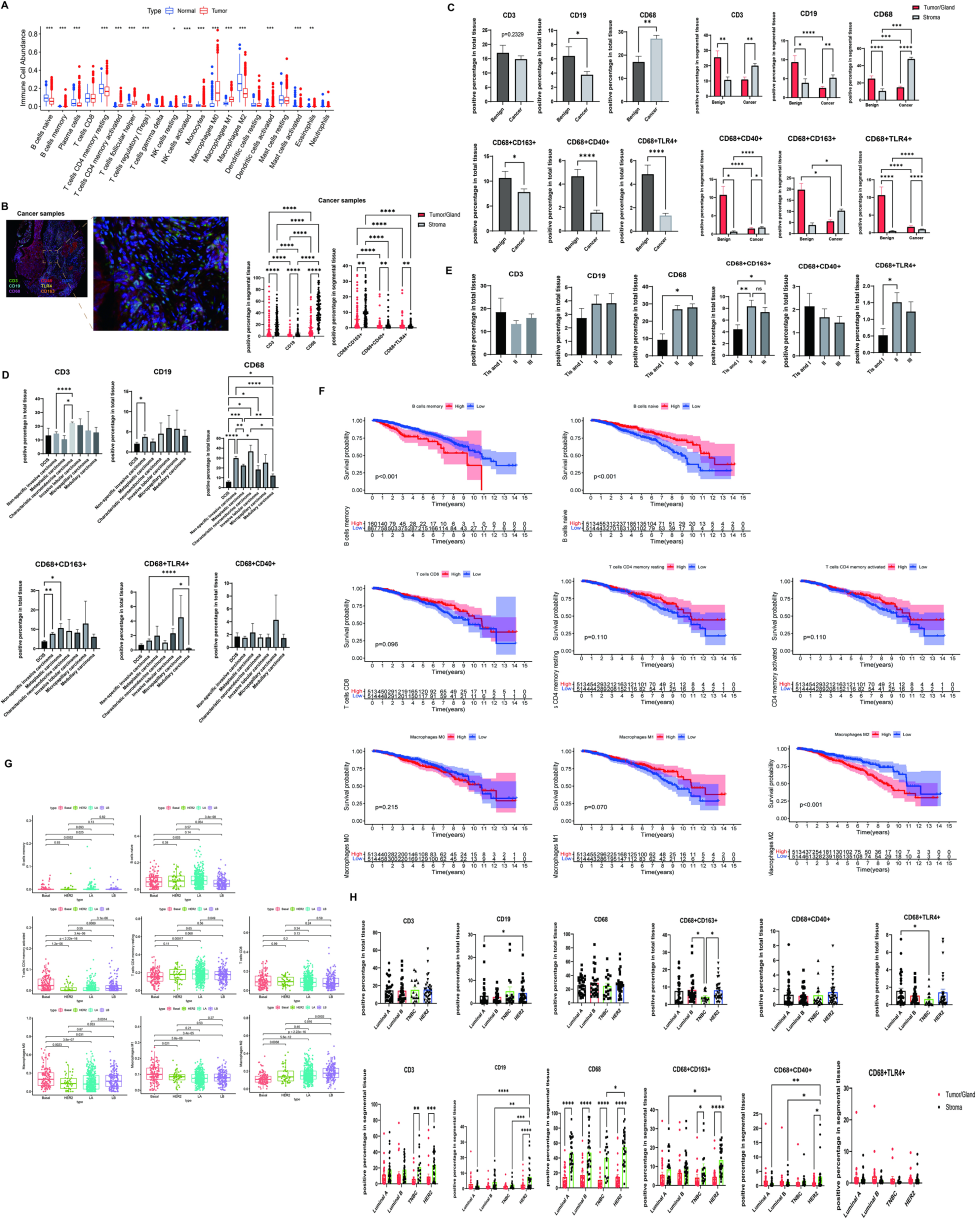
The immune characteristics of the breast cancer microenvironment. **(A)** Different immune cells in breast cancer from TCGA database. **(B)** Opal-IHC analysis for T cells, B cells and macrophages in breast cancer samples. The results indicate that the infiltration level of CD68+ macrophages in the breast cancer microenvironment is significantly higher than that of CD3+ T cells and CD19+ B cells, and is predominantly concentrated in the stromal regions. Among CD68+ macrophages, the CD163+ subtype constitutes the majority and is also mainly localized in the stromal areas. **(C)** Comparison of the proportional characteristics of immune cell infiltration between benign and malignant tissues. Benign N = 19, cancer N = 114. **(D)** The immune phenotype analysis among diverse pathological types of malignant tissues. **(E)** The immune phenotype analysis among diverse clinical stages. **(F, G)**. The immune cell abundance of diverse subtypes of B cells, T cells, and macrophages among diverse molecular types using the TCGA database. **(H)** The immune cell abundance of diverse subtypes of B cells, T cells, and macrophages among diverse molecular types. The data are evaluated with the 2-way and one-way ANOVA with the Tukey test or 2-tailed unpaired t-test. *p<0.05; **p<0.01; ***p<0.005; ****p<0.001.

To delineate the immune landscape of breast cancer, we first analyzed TCGA data, which confirmed a complex infiltrate comprising diverse T cells, B cells, NK cells, and macrophage subsets (**Figure 2A**). We then performed an in-depth spatial analysis using multiplex fluorescence immunohistochemistry (mIHC) on a tissue microarray encompassing 151 clinical samples, enabling simultaneous quantification of CD3^+^ T cells, CD19^+^ B cells, and distinct macrophage subpopulations (CD68^+^CD163^+^ M2-like, CD68^+^TLR4^+^ M1-like, CD68^+^CD40^+^ TAM) within the tumor microenvironment (**Figure 2B**).

Comparative analysis revealed that malignant breast tissues exhibited significantly greater overall immune cell infiltration compared to benign tissues, with a notable redistribution of macrophages from periglandular areas into the tumor stroma (**Figure 2C**). When stratified by pathology, ductal carcinoma *in situ* (DCIS) showed the lowest level of macrophage infiltration, including CD68^+^TLR4^+^ macrophages, whereas T and B cell levels remained comparable across groups (**Figure 2D**). Importantly, progression from DCIS to Stage II invasive carcinoma was associated with a significant increase in the abundance of CD68^+^CD163^+^ M2-like macrophages (**Figure 2E**). The clinical relevance of this observation was underscored by multivariate Cox regression analysis, which identified M2-like macrophage density as an independent prognostic factor for poor overall survival in our cohort (**Table 1**). This finding was corroborated by Kaplan-Meier analysis of TCGA data, which further demonstrated a significant association between high M2 macrophage abundance and poor patient prognosis (**Figure 2F**).

**Table 1:** Survival analysis of M2 in breast cancer.

Factor	HR	HR.95L	HR.95H	P-value
Age	1.033345241	1.018216622	1.04869864	1.31E-05
Stage	1.584536401	0.961604782	2.611005743	0.070868213
T	1.012817097	0.757300474	1.35454619	0.931579698
N	1.219694095	0.919334278	1.618185814	0.168552225
M	1.671368951	0.738795096	3.7811217	0.217517213
Gender	0.530751065	0.073460156	3.834686847	0.530116021
Macrophage M2	10.56678119	2.205083697	50.6361118	0.003187368

Cox Multivariate analysis model is applied to investigate the indicated values of M2 together with other clinical factors. T, tumor size; N, Lymph node metastasis; M, distant metastasis.

Crucially, when we stratified the immune landscape by molecular subtype, a distinct pattern emerged. Both our mIHC data and independent validation using the TCGA cohort revealed that TNBC subtype harbored the lowest abundance of CD68+CD163+ macrophages compared to all other subtypes (Luminal A, Luminal B, and HER2+). This reduction in CD163+ infiltration was consistently observed in both the global tumor area and within specific stromal compartments upon segmentation analysis (**Figures 2G, H**).

Collectively, these data delineate a unique and paradoxical immune signature for TNBC. Despite its aggressive clinical behavior, its microenvironment is characterized by a relative paucity of typically pro-tumorigenic CD163^+^ macrophages. This profile suggests an altered balance in the immune response, potentially favoring a hyper-inflammatory state that may underpin the aggressiveness of TNBC. To investigate the mechanism underlying the low infiltration of CD163^+^ macrophages in TNBC, we next established an *in vitro* co-culture model to assess the effect of soluble factors secreted by TNBC cells on macrophage polarization.

### 3.2 Conditioned medium from TNBC cells inhibits CD163^+^ macrophage polarization *in vitro*

To investigate how TNBC shapes a pro-tumorigenic immune microenvironment, we established a co-culture system to assess the impact of cancer cell-secreted factors on macrophage polarization. We first generated and validated a robust *in vitro* model by differentiating THP-1 monocytes into M0 macrophages using PMA, followed by induction into canonical M1 or M2 phenotypes, as confirmed by morphology, surface marker expression (CD68, CD163), gene expression profiles, and functional phagocytosis assays (**Supplementary Figure 2**).

**Figure 3:**
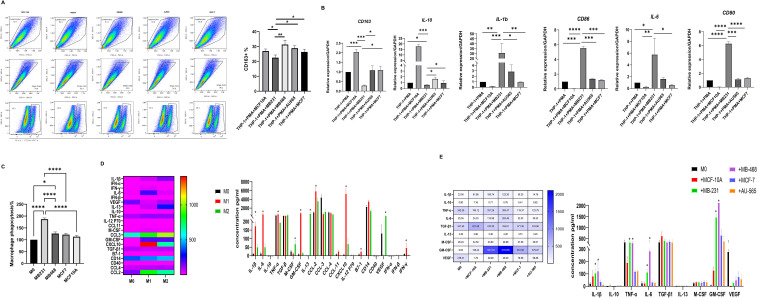
TNBC promoted macrophage polarization to an M1-like subtype. **(A)** FC analysis of macrophages cultured with conditioned medium from diverse cell subtypes compared to M0, and the gate strategy is SSC-A/FSC-A to exclude debris, SSC-A/SSC-H to select singlet, and SSC-A/CD163 to gate CD163+ population. N = 5 **(B)** qRT-PCR is used to evaluate the expression levels of diverse classic M1/M2 markers. N = 3. **(C)** The analysis of phagocytosis ability. N = 3. **(D, E)** Flow-based multi-indicator enzyme immunoassay to detect 21 inflammatory factors is used to evaluate the immunoprofiles among M0, M1, and M2 **(D)** and among different CM-M0 groups **(E)** N = 8. The data are evaluated with the 2-way and one-way ANOVA with the Tukey test or 2-tailed unpaired t-test. *p<0.05; **p<0.01; ***p<0.005; ****p<0.001.

We next collected conditioned medium (CM) from a panel of breast cell lines—representing normal epithelium (MCF-10A), TNBC (MDA-MB-231 and MDA-MB-468), and other molecular subtypes (MCF-7, AU-565)—and co-cultured with M0 macrophages. Flow cytometric analysis revealed that CM from MDA-MB-231 cells, a representative TNBC line, was the most potent in suppressing the acquisition of the CD163+ phenotype (**Figure 3A**). Concordantly, macrophages treated with MDA-MB-231-CM exhibited a transcriptional profile skewed towards a pro-inflammatory state, characterized by significant upregulation of IL-1β, IL-6, CD80, and CD86, and concomitant downregulation of IL-10 and CD163 (**Figure 3B**). Functionally, these macrophages also demonstrated enhanced phagocytic activity, a hallmark of M1-like macrophages (**Figure 3C**).

To obtain a comprehensive inflammatory profile, we analyzed 21 soluble factors. As expected, canonical M1 macrophages secreted high levels of IL-1β, IL-6, TNF-α, GM-CSF, CCL2, CXCL10, and IFN-γ compared to M0 and M2 controls (**Figure 3D**). Mirroring this signature, macrophages conditioned with CM from TNBC cells (MDA-MB-231 and MDA-MB-468) secreted significantly higher amounts of IL-1β, TNF-α, IL-6, and most notably, GM-CSF, compared to those treated with CM from non-TNBC cells (**Figure 3E**).

In summary, these data demonstrate that soluble factors secreted by TNBC cells, particularly MDA-MB-231, potently drive macrophage polarization towards a pro-inflammatory, M1-like state, while concurrently inhibiting alternative M2 activation.

### 3.3 Identification and validation of an aberrantly activated IRF7-CH25H-25HC pathway in TNBC

**Figure 4:**
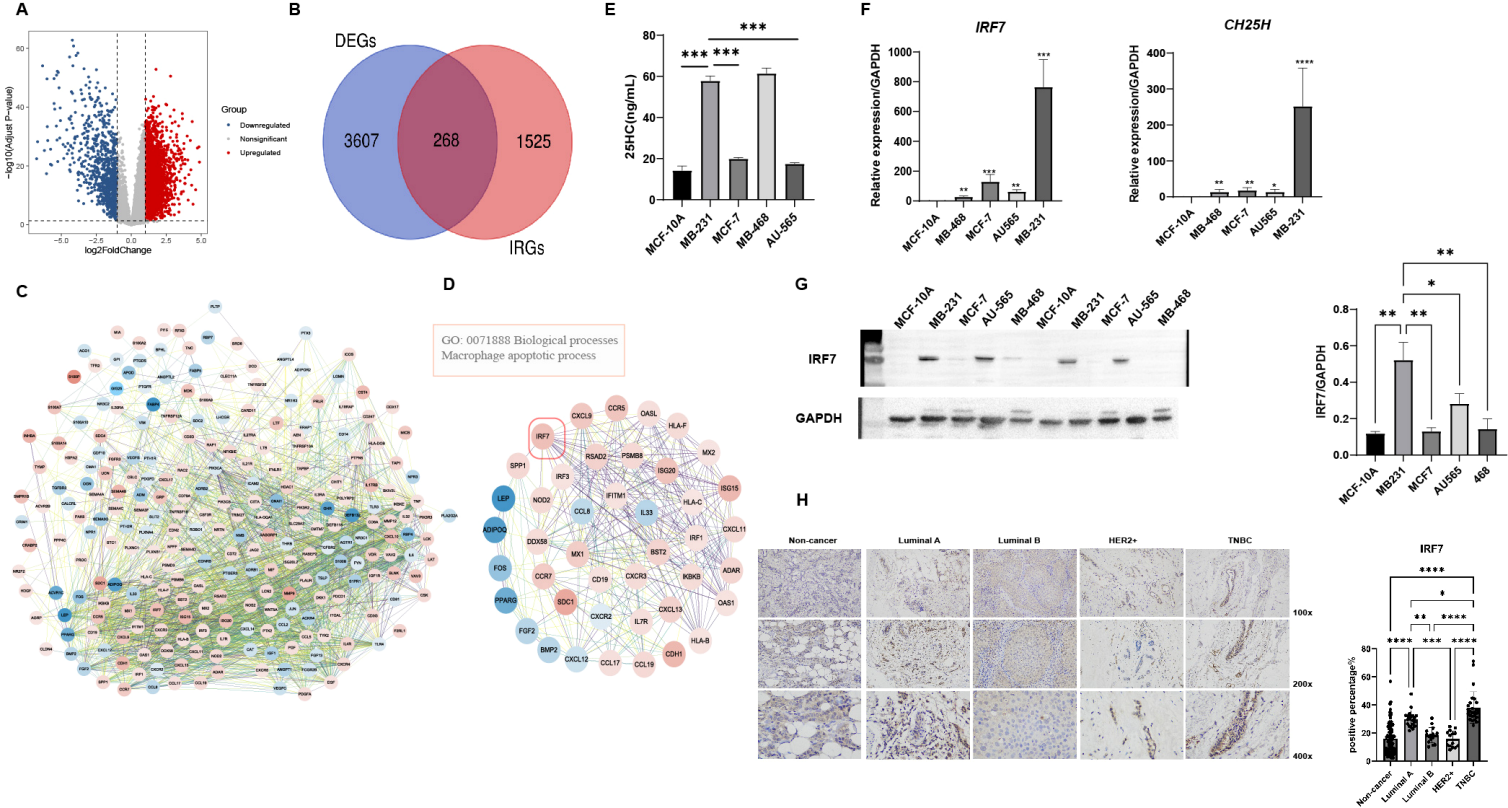
Statuses of IRF7/CH25H/25HC pathway in diverse molecular types. **(A)** DEG identification based on the GSE42568 dataset. **(B)** The overlapping genes between DEGs and IRGs. **(C)** PPI network constructed by 268 overlapping genes. **(D)** The most significant module sorted out from the PPI network and potential macrophage-related biological process enriched by the 1 framed gene. **(E)** The different concentrations of 25HC among diverse cell lines. N = 3. **(F)** qRT-PCR analysis of *IRF7* and *CH25H* among diverse cell lines. N = 6. **(G)** WB analysis of IRF7 among diverse cells. N = 3. **(H)** IHC analysis of the expression of IRF7 among diverse molecular types using BRCA samples. The total number of samples was 107 (HER2 positive n=15; Luminal A n=19; Luminal B n=17; TNBC n=24; non-cancer n=32). The data are evaluated with the 2-way and one-way ANOVA with the Tukey test or 2-tailed unpaired t-test. *p<0.05; **p<0.01; ***p<0.005; ****p<0.001.

To elucidate the mechanism underlying TNBC-driven macrophage polarization, we employed a bioinformatics approach. Differential expression analysis of breast cancer transcriptomes (GSE42568) identified numerous dysregulated genes (**Figure 4A**). Intersection with a database of immune-related genes (IRGs) yielded 268 candidates potentially involved in immune modulation (**Figure 4B**). Protein-protein interaction network and functional enrichment analyses of these overlapping genes highlighted a cluster associated with interferon signaling and macrophage regulation, with Interferon Regulatory Factor 7 (IRF7) emerging as a central node (**Figures 4C, D**).

Given the documented role of the IRF7-CH25H-25HC axis in innate immunity, we hypothesized it might be operative in TNBC. We first quantified the end product, 25-hydroxycholesterol (25HC), in conditioned media. ELISA confirmed that TNBC cell lines, particularly MDA-MB-231, secreted significantly higher levels of 25HC compared to normal breast epithelial cells (MCF-10A) or other molecular subtypes (**Figure 4E**). Correspondingly, qRT-PCR analysis revealed a pronounced upregulation of both *IRF7* and its transcriptional target, Cholesterol-25-Hydroxylase (*CH25H*), in TNBC cells, with MDA-MB-231 exhibiting the most substantial increase (**Figure 4F**). This elevated IRF7 expression was confirmed at the protein level by western blotting (**Figure 4G**). Importantly, this pathway activation was clinically relevant: immunohistochemical staining of patient-derived breast cancer tissues demonstrated significantly higher IRF7 protein expression in TNBC samples compared to other subtypes (**Figure 4H**).

Collectively, these findings from bioinformatic prediction, *in vitro* models, and clinical specimens converge to identify the IRF7-CH25H-25HC axis as a specifically and aberrantly activated pathway in TNBC.

### 3.4 25HC differentially regulates macrophage polarization and mediates the TNBC effect

**Figure 5:**
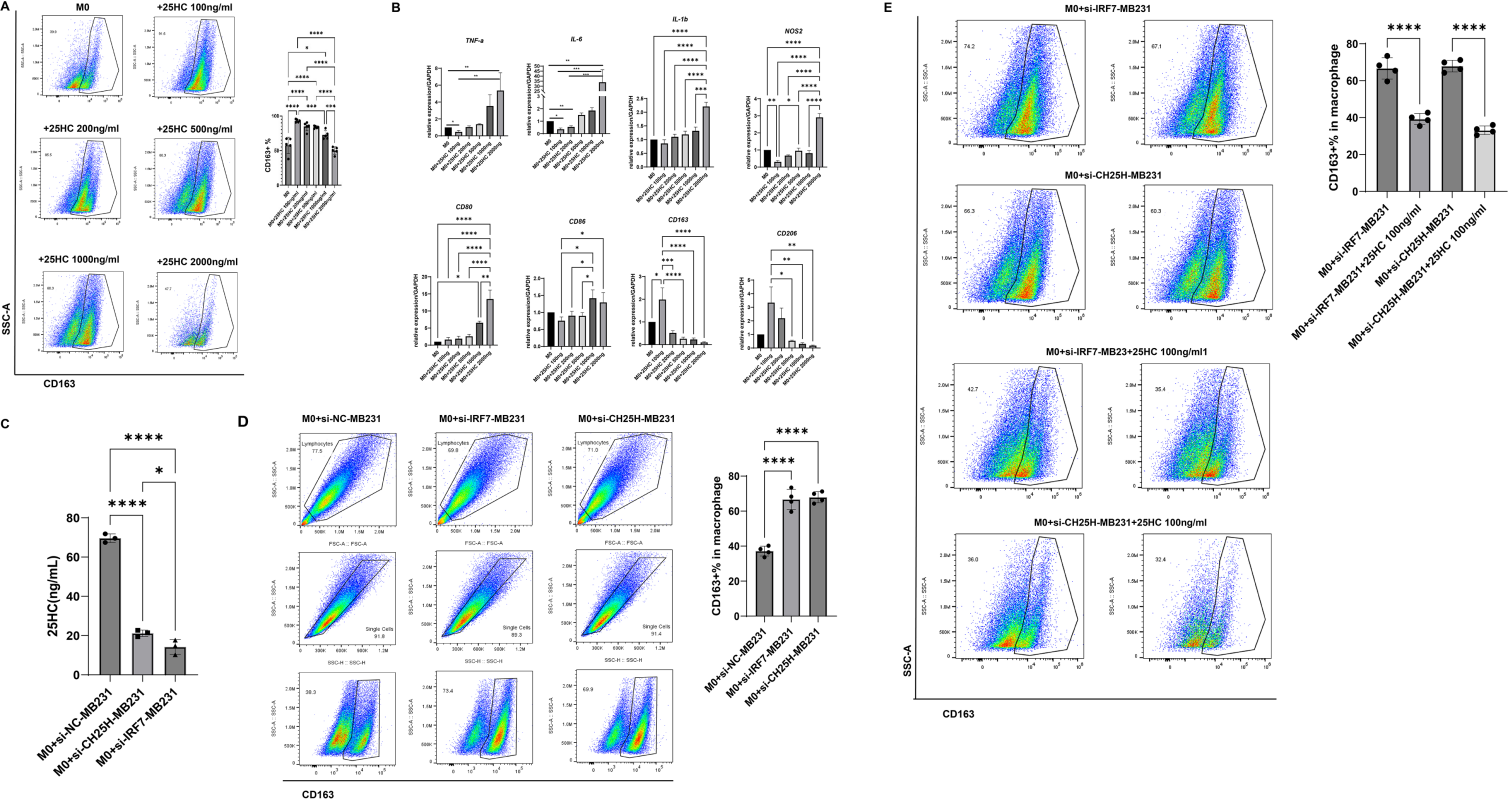
Roles of IRF7/CH25HC/25HC in regulating macrophage polarization. **(A)** FC evaluated the role of diverse concentrations of 25HC in modulating macrophage polarization. N = 5. **(B)** qRT-PCR is used to evaluate the expression levels of diverse M1/M2 markers among diverse concentrations of 25HC groups. N = 4. **(C)** Effect of knockdown of *IRF7* and *CH25H* expression levels in MB231 cells on M2 ratio using FC. N = 4. **(D)** Effect of knocking down the expression levels of *IRF7* and *CH25H* on the end product 25HC using ELISA. N = 3. **(E)** Additional 25HC reverses the altered ability of MB231 to regulate macrophage polarization. N = 4. The data are evaluated with the 2-way and one-way ANOVA with the Tukey test or 2-tailed unpaired t-test. *, p<0.05; **, p<0.01; ***, p<0.005; ****, p<0.001.

Having established that TNBC cells produce high levels of 25HC, we sought to determine its direct effect on macrophages. Treatment of M0 macrophages with purified 25HC revealed a concentration-dependent, bidirectional impact on polarization. Low concentrations of 25HC promoted an M2-like phenotype, evidenced by increased CD206+ cell frequency and elevated expression of *CD163* and *CD206*. In stark contrast, high concentrations of 25HC potently induced an M1-like state, characterized by reduced *CD206* expression and significant upregulation of pro-inflammatory markers including *TNF-α, IL-6, IL-1β, NOS2*, *CD80*, and *CD86* (**Figures 5A, B**). This demonstrates that 25HC itself is a potent and context-dependent regulator of macrophage fate, and the high levels found in the TNBC microenvironment favor a pro-inflammatory shift.

To definitively link the observed TNBC-driven polarization to this pathway, we performedloss-of-function experiments. Knockdown of either *IRF7* or *CH25H* inMDA-MB-231 cells (**Supplementary Figures 3A, B**) significantly reduced the secretion of 25HC (**Figure 5C**). Consequently, the conditioned medium from these knockdown cells lost the ability to suppress M2 polarization, instead leading to a marked increase in the proportion of CD163+ macrophages (**Figure 5D**). Crucially, this effect was reversible: supplementing the knockdown cell-conditioned medium with exogenous 25HC (100 ng/mL) fully restored its capacity to inhibit CD163+ macrophage generation (**Figure 5E**).

Taken together, these data establish a direct causal relationship. They demonstrate that the IRF7-CH25H-25HC axis is both necessary and sufficient for TNBC cells to dictate macrophage polarization towards an M1-like phenotype, with the concentration of the metabolite 25HC acting as the critical effector.

### 3.5 The IRF7-25HC axis promotes TNBC tumor growth and shapes the immune microenvironment *in vivo*

We next investigated the functional consequences of elevated 25HC on TNBC cells themselves. Strikingly, while 25HC exerted concentration-dependent inhibitory effects on the viability of non-TNBC cell lines (MCF-7, AU-565) and normal mammary epithelial cells (MCF-10A), it enhanced the proliferation of MDA-MB-231 cells, as measured by CCK-8 assay (**Figure 1A**). Cell cycle analysis indicated that this pro-proliferative effect was associated with a reduction in apoptosis at higher 25HC concentrations (**Figure 1B**). This TNBC-specific growth promotion suggests that 25HC not only remodels the immune microenvironment but also functions as an autocrine pro-tumorigenic factor.

To validate the pathophysiological relevance of the IRF7-25HC axis, we turned to *in vivo* models. Knockdown of IRF7 in MDA-MB-231 cells significantly impaired tumor growth in immunodeficient nude mice, confirming the cell-autonomous role of this pathway in supporting TNBC proliferation (**Figure 1C**). Crucially, the growth defect in IRF7-knockdown tumors was partially but significantly rescued by systemic administration of 25HC, demonstrating that the metabolite is a key downstream effector of IRF7-driven tumor growth (**Figure 1D**).

To assess the impact on the intact tumor immune microenvironment, we employed a syngeneic model using Irf7-knockdown 4T1 cells in immunocompetent mice. Consistent with the xenograft data, Irf7 knockdown potently suppressed tumor growth (**Figure 1E**). Flow cytometric analysis of these tumors revealed a significant increase in the proportion of CD163+ macrophages, providing direct *in vivo* evidence that silencing the IRF7-25HC axis in TNBC cells alleviates the suppression of CD163+ polarization within the TME (**Figure 1F**).

In summary, these *in vivo* findings demonstrate that the aberrantly activated IRF7-25HC axis in TNBC exerts a dual pro-tumorigenic function: it cell-autonomously enhances tumor cell growth, and it non-cell-autonomously sculpts an immunosuppressive microenvironment by restraining CD163+ macrophage polarization.

## 4 Discussion

The dynamic interplay between cancer cells and the tumor immune microenvironment (TIME) is a critical determinant of disease progression and therapeutic outcome (8, 9). While T cell-centric therapies have revolutionized oncology (31–34), targeting tumor-associated macrophages (TAMs)—the most abundant immune population in breast cancer—remains a formidable challenge due to their profound heterogeneity and plasticity (16, 17). The functional polarization of TAMs is exquisitely sensitive to local environmental cues (35–39), yet the specific metabolic signals within the TIME that dictate macrophage fate, particularly in aggressive subtypes like TNBC, are poorly defined. In this study, we identify the oxysterol metabolite 25-hydroxycholesterol (25HC) as a novel, TNBC-specific immunometabolic cue that directly regulates macrophage polarization. Furthermore, we unveil a previously unrecognized autocrine role for 25HC in promoting TNBC cell proliferation, revealing a dual-pathway mechanism through which the IRF7-CH25H-25HC axis drives TNBC pathogenesis.

Our integrated analysis of clinical specimens and bioinformatics data first revealed a unique immune contexture in TNBC, characterized by a significantly lower infiltration of CD163+CD68+ M2-like macrophages compared to other molecular subtypes, alongside evidence of T cell activation. This apparent paradox—where the most aggressive subtype exhibits fewer typically pro-tumorigenic M2 macrophages—aligns with the emerging concept that chronic inflammation and immune activation can themselves be drivers of tumorigenesis (19). It also suggests that the failure of some immunotherapies in TNBC may stem from an oversimplified focus on reversing immune suppression, rather than addressing underlying hyper-inflammatory mechanisms.

Our discovery positions the IRF7-CH25H-25HC axis as a central pathway linking TNBC cell-intrinsic oncogenic signaling to immune reprogramming. While 25HC is recognized for its antiviral and broad immunomodulatory functions (21), and IRF7 is a well-established master regulator of interferon responses (40), their role in cancer, particularly in shaping the TIME, has been obscure. Our bioinformatic screening pinpointed IRF7 as a key regulator in TNBC, leading us to uncover its aberrant activation and consequent overproduction of 25HC. The bidirectional regulation of macrophage polarization by 25HC concentration provides a mechanistic explanation for the TNBC-specific M2 suppression we observed. It is plausible that the chronically high 25HC in the TNBC niche saturates signaling pathways in TAMs, potentially desensitizing or altering the output of their intrinsic IRF7 pathway—a phenomenon supported by studies showing that IRF7 activity is critical for setting inflammatory thresholds in myeloid cells (41–46). Our previous studies in the mammalian testicular microenvironment revealed that the interferon-steroid regulatory pathway IRF7/25HC can alter the subtypes of tissue-resident macrophages, primarily manifested as changes in MHC expression levels. Other studies have also demonstrated that macrophages, as members of antigen-presenting cells, significantly co-regulate the downstream functions of both T and B cells through their MHC expression levels. Thus, our findings further confirm that IRF7/25HC in the TNBC microenvironment markedly modifies the proportion of CD163+ macrophages and may simultaneously modulate MHC expression status, thereby influencing downstream activation pathways of antigen-presenting cells (APCs). This creates a feed-forward loop where TNBC cells, through constitutive IRF7 activation, generate a 25HC-rich milieu that locks infiltrating macrophages into a pro-inflammatory, M1-like state, thereby subverting a key adaptive immune resistance mechanism.

Perhaps even more striking is our finding that 25HC acts as an autocrine growth factor for TNBC cells. This cell-type-specific proliferative effect reveals a parallel, non-immunological function of this pathway that directly contributes to TNBC aggressiveness. The contrasting sensitivity of non-TNBC lines to 25HC-induced suppression suggests that TNBC cells have adapted to leverage this metabolite for their own advantage, possibly through the differential expression of receptors (e.g., LXRs) or downstream survival pathways. This dual role of the IRF7-25HC axis—fueling both cell-autonomous proliferation and non-cell-autonomous immunomodulation—represents a novel paradigm in TNBC biology, illustrating how a single metabolic pathway can co-opt fundamental immunity mechanisms for multifaceted tumor promotion.

Our finding that TNBC exhibits reduced CD163+ macrophage infiltration despite its aggressive behavior aligns with a recent comprehensive review on TAMs in TNBC, which notes that while TAMs in TNBC are predominantly polarized toward an M2-like phenotype under the influence of cytokines such as IL-10 and TGF-β, the functional outcomes of TAM polarization are highly context-dependent and can vary across different stages of tumor progression (47). Consistent with this, our group has recently reported that TNBC remodels the tissue immune microenvironment by regulating embryonic-derived tissue-resident macrophage polarization through the IRF7/CH25H/25HC pathway (48). Notably, a recent study in melanoma revealed an opposing role for 25HC: lymphatic endothelial cell-derived 25HC inhibits PPAR-γ in intra-tumoral macrophages, promoting their conversion into pro-inflammatory myeloid cells that support effector T cell function and correlate with better patient survival and response to immunotherapy (26). This striking contrast—where 25HC promotes anti-tumor immunity in melanoma but facilitates tumor progression in TNBC—underscores the context-dependent nature of oxysterol immunomodulation and highlights the importance of understanding tumor type-specific metabolic-immune interactions.

## 5 Clinical implications and future perspectives

Our study, traversing from clinical bioinformatics to *in vivo* validation, delineates a coherent model in which TNBC cells co-opt the IRF7-CH25H-25HC axis to foster a tumor-promoting ecosystem via dual autocrine and paracrine mechanisms. This model provides a metabolic-immune framework to explain the aggressive and therapy-resistant nature of TNBC.

From a translational standpoint, our findings nominate the IRF7-CH25H-25HC axis as a compelling therapeutic target. Inhibiting this pathway—for instance, by targeting CH25H enzymatic activity or neutralizing 25HC—holds the potential to simultaneously dampen TNBC cell proliferation and reverse the pro-tumorigenic immune contexture, offering a synergistic approach to combination therapy. This strategy might be particularly effective when paired with existing immunotherapies, such as immune checkpoint blockade, by mitigating the underlying inflammatory drive that may limit their efficacy in TNBC (30, 49).

While our findings demonstrate that the IRF7-CH25H-25HC axis significantly influences the TNBC immune microenvironment, it is important to recognize that the TME is a complex ecosystem involving multiple cell types beyond macrophages. Cancer-associated fibroblasts (CAFs), endothelial cells, T cells, B cells, NK cells, myeloid-derived suppressor cells (MDSCs), and dendritic cells all contribute to the overall immune landscape. Recent reviews have highlighted the intricate crosstalk between TAMs and other TME components in TNBC (14), including the role of TAMs in promoting angiogenesis, suppressing T cell function, and remodeling the extracellular matrix. In our syngeneic 4T1 model, we observed that Irf7 knockdown not only increased CD163+ macrophage infiltration but also altered CD8+ T cell infiltration in the TME, suggesting that the effects of this axis extend beyond macrophages to broader immune reprogramming. The mechanistic basis of this broader immune modulation—whether it occurs directly through 25HC-mediated effects on T cells or indirectly through macrophage-T cell crosstalk—requires further investigation. Nevertheless, our results indicate that the IRF7-CH25H-25HC axis represents a critical node in the TNBC immune regulatory network, and its modulation may have pleiotropic effects on the TME.

Several important questions remain for future investigation. First, the precise receptor and downstream signaling mechanisms by which 25HC exerts its bidirectional effects on macrophages and its proliferative effects on TNBC cells require elucidation; the liver X receptors (LXRs) and other potential candidates warrant examination. Second, the upstream drivers of constitutive IRF7 activation in TNBC, such as chronic activation of the cGAS-STING pathway or other innate immune sensors, represent a critical area for future study. Finally, validating the therapeutic efficacy of targeting this axis in patient-derived organoids and humanized mouse models will be an essential step toward clinical translation.

In conclusion, we have identified an oncogenic and immunomodulatory pathway that is aberrantly activated in TNBC. The IRF7-CH25H-25HC axis serves as a linchpin connecting tumor cell metabolism to immune evasion and autonomous growth, revealing new vulnerabilities for combating this challenging disease.

## Data Availability

The datasets presented in this study can be found in online repositories. The names of the repository/repositories and accession number(s) can be found in the article/**Supplementary Material**.

## Supplementary material

Supplementary Figure 1 The process of multicolor immunofluorescence immunohistochemical analysis and the overall characteristics of the breast cancer immune microenvironment. **(A)** IHC staining of each antibodies and multiple fluorescent dyes were coupled as follows: Opal 520 for anti-CD3 (green), Opal 540 for anti-CD68 (blue), Opal 570 for anti-CD163 (purple), Opal 620 for anti-CD19 (pink), Opal 650 for anti-CD40 (orange), and Opal 690 for anti-TLR4 (red) as showed. Each sample was analyzed individually with 6 phenotype markers in total or segmental areas as displayed. **(B)** The immune cell phenotypes analysis flow chart with InForm software. Images were captured at 200x magnification with AKOYA-Vectra 3, and were analyzed with InForm 2.1 software.

Supplementary Figure 2 Establishment of an *in vitro* macrophage induction model. **(A)** THP-1-induced macrophage (PMA-induced) morphology is observed under a light microscope. **(B)** FC verified the expression levels of the surface markers CD11c, CD11b, and CD68 in macrophages and monocytes. N = 3. **(C)** IF verifies the expression status of CD11c, CD206, and CD68. N = 3. **(D)** qRT-PCR is used to measure the expression levels of macrophage markers. N = 3. **(E)** FC assay is used to measure CD206+ percentages among M0, M1, and M2 subtypes. N = 9. **(F)** qRT-PCR is used to measure the expression levels of macrophage subtype markers. N = 4. **(G)** is used to verify CD206 expression. N = 6. H) Analysis of phagocytic ability. N = 3. The data are evaluated with the 2-way and one-way ANOVA with the Tukey test or 2-tailed unpaired t-test. *p<0.05; **p<0.01; ***p<0.005; ****p<0.001.

Supplementary Table 1 The list of PCR primers used in this study.

Supplementary Table 2 List of breast cancer tissues with molecular classification according to National Comprehensive Cancer Network guideline in 2021
