# Supplementary figures and images for: 25HC regulates the polarization of CD163+ macrophages in the immune microenvironment of triple-negative breast cancer through the interferon pathway
Source: Front Immunol. 2026 May 28;17:1812056. doi: 10.3389/fimmu.2026.1812056 (PMC13253280; doi:10.3389/fimmu.2026.1812056)

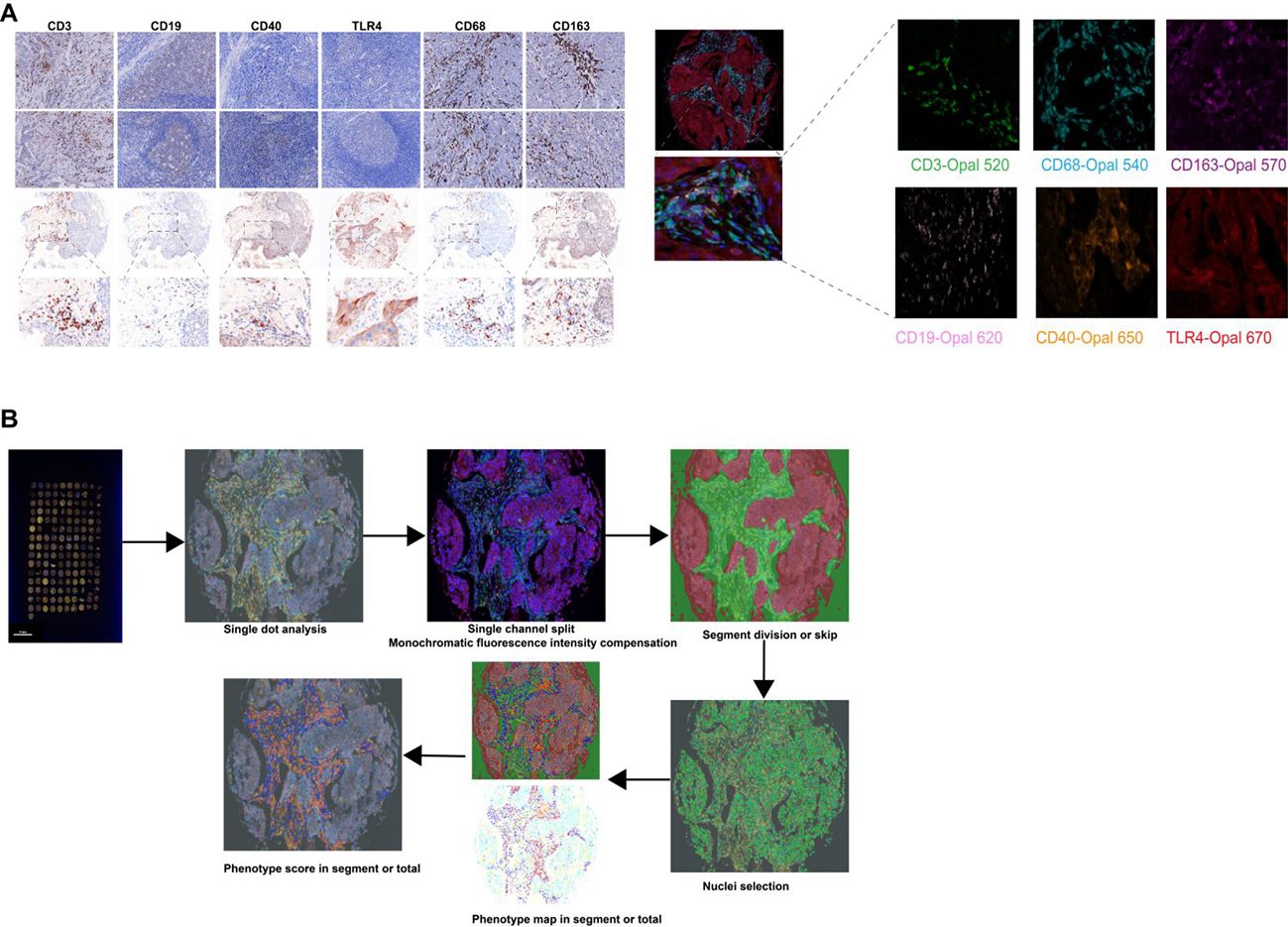

Supplement: Supplementary Figure 1 — The process of multicolor immunofluorescence immunohistochemical analysis and the overall characteristics of the breast cancer immune microenvironment. (A) IHC staining of each antibodies and multiple fluorescent dyes were coupled as follows: Opal 520 for anti-CD3 (green), Opal 540 for anti-CD68 (blue), Opal 570 for anti-CD163 (purple), Opal 620 for anti-CD19 (pink), Opal 650 for anti-CD40 (orange), and Opal 690 for anti-TLR4 (red) as showed. Each sample was analyzed individually with 6 phenotype markers in total or segmental areas as displayed. (B) The immune cell phenotypes analysis flow chart with InForm software. Images were captured at 200x magnification with AKOYA-Vectra 3, and were analyzed with InForm 2.1 software. [file Image1.jpeg]

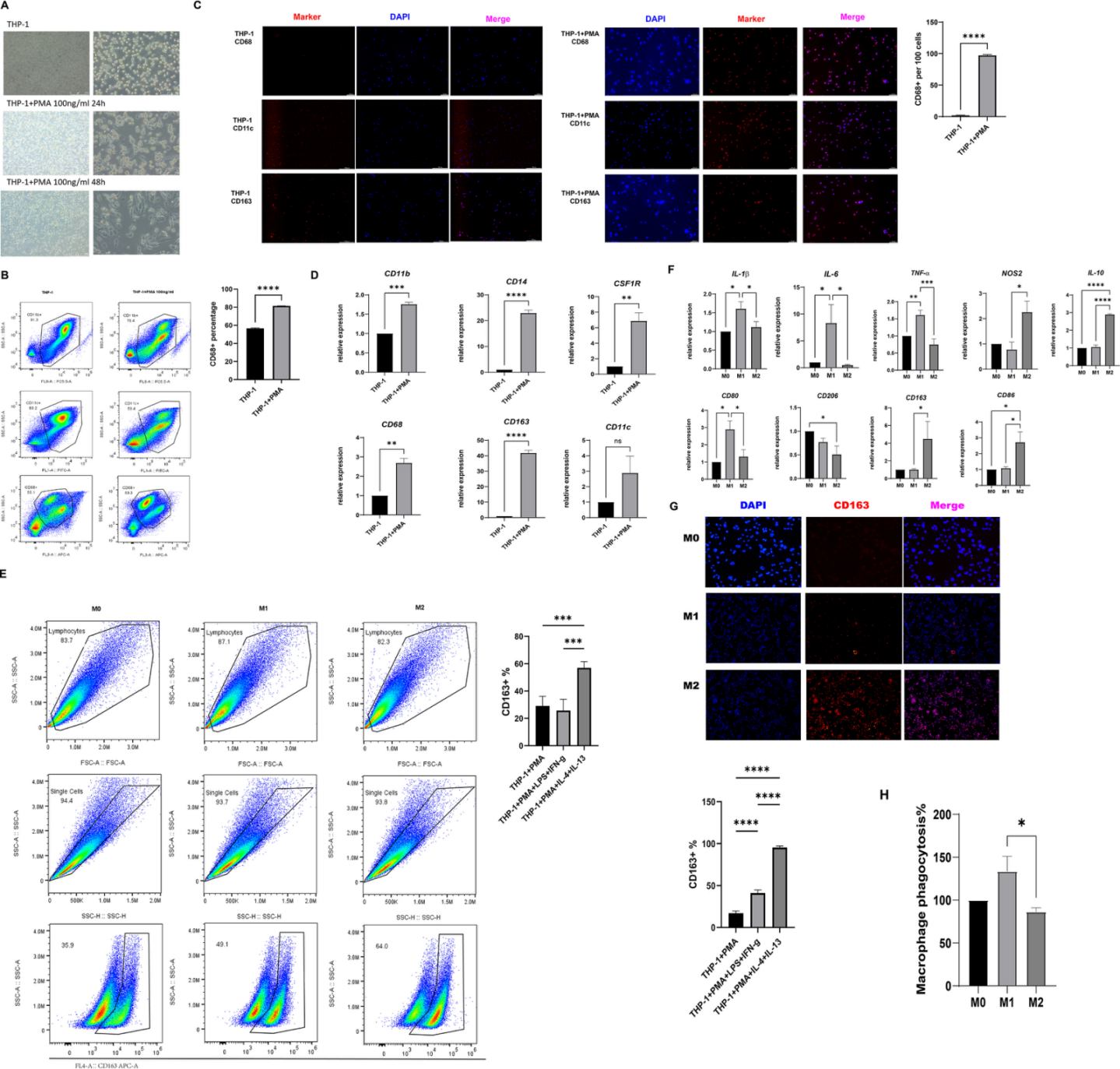

Supplement: Supplementary Figure 2 — Establishment of an in vitro macrophage induction model. (A) THP-1-induced macrophage (PMA-induced) morphology is observed under a light microscope. (B) FC verified the expression levels of the surface markers CD11c, CD11b, and CD68 in macrophages and monocytes. N = 3. (C) IF verifies the expression status of CD11c, CD206, and CD68. N = 3. (D) qRT-PCR is used to measure the expression levels of macrophage markers. N = 3. (E) FC assay is used to measure CD206+ percentages among M0, M1, and M2 subtypes. N = 9. (F) qRT-PCR is used to measure the expression levels of macrophage subtype markers. N = 4. (G) is used to verify CD206 expression. N = 6. H) Analysis of phagocytic ability. N = 3. The data are evaluated with the 2-way and one-way ANOVA with the Tukey test or 2-tailed unpaired t-test. *p<0.05; **p<0.01; ***p<0.005; ****p<0.001. [file Image2.jpeg]
