# Supplementary material for: 25HC regulates the polarization of CD163+ macrophages in the immune microenvironment of triple-negative breast cancer through the interferon pathway
Source: Front Immunol. 2026 May 28;17:1812056. doi: 10.3389/fimmu.2026.1812056 (PMC13253280; doi:10.3389/fimmu.2026.1812056)
Supplement: Supplementary Table 2 — List of breast cancer tissues with molecular classification according to National Comprehensive Cancer Network guideline in 2021 [file Table2.docx]

| **Location** | **Age** | **Gender** | **Pathological diagnosis** | **Grade** | **TNM** | **Stage** | **Tissue Type** | **Tumor Size** | **Lymph**  **node**  **metastasis** | **IHC1** | **IHC2** | **IHC3** | **IHC4** | **IHC5** |
| --- | --- | --- | --- | --- | --- | --- | --- | --- | --- | --- | --- | --- | --- | --- |
| A1 | 45 | Female | Adenosis of breast ，From  G6 | - | - | - | Para-cancer | - | - | ER: + | PR: + + | Her2: 1 + | Ki-67: <1% | - |
| A2 | 47 | Female | Breast Tissue ，From H7 | - | - | - | Para-cancer | - | - | ER: + + + | PR: + + | Her2: 3 + | Ki-67: 8% | - |
| A3 | 40 | Female | Breast Tissue ，From C3 | - | - | - | Para-cancer | - | - | ER: + | PR: + + | Her2: 1 + | Ki-67: 5% | - |
| A4 | 50 | Female | Breast Tissue ，From C9 | - | - | - | Para-cancer | - | - | ER: + + + | PR: + + + | Her2: * | Ki-67: * | - |
| A5 | 56 | Female | Breast Tissue ，From H1 | - | - | - | Para-cancer | - | - | ER: + | PR: + + | Her2: 0 | Ki-67: 5% | - |
| A6 | 88 | Female | Breast Tissue ，From H5 | - | - | - | Para-cancer | - | - | ER: + | PR: + | Her2: 0 | Ki-67: <1% | - |
| A7 | 53 | Female | Breast Tissue ，From H8 | - | - | - | Para-cancer | - | - | ER: + + + | PR: - | Her2: 0 | Ki-67: <1% | - |
| A8 | 84 | Female | Breast Tissue ，From H2 | - | - | - | Para-cancer | - | - | ER: + + | PR: - | Her2: 0 | Ki-67: <1% | - |
| A9 | 43 | Female | Breast Tissue ，From I4 | - | - | - | Para-cancer | - | - | ER: + + | PR: + + | Her2: 0 | Ki-67: <1% | - |
| A10 | 51 | Female | Adenosis of breast ，From  E3 | - | - | - | Para-cancer | - | - | ER: + + + | PR: + | Her2: 1 + | Ki-67: <1% | - |
| A11 | 26 | Female | chronic mastitis | - | - | - | Benign | - | - | ER: + + + | PR: - | Her2: 1 + | Ki-67: <1% | - |
| A12 | 57 | Female | chronic mastitis | - | - | - | Benign | - | - | ER: + + | PR: - | Her2: 0 | Ki-67: 2% | - |

| A13 | 62 | Female | chronic mastitis | - | - | - | Benign | - | - | ER: + + + | PR: - | Her2: 0 | Ki-67: <1% | - |
| --- | --- | --- | --- | --- | --- | --- | --- | --- | --- | --- | --- | --- | --- | --- |
| A14 | 47 | Female | chronic mastitis | - | - | - | Benign | - | - | ER: + | PR: - | Her2: 0 | Ki-67: <1% | - |
| A15 | 28 | Female | Adenosis of breast | - | - | - | Benign | - | - | ER: + + + | PR: + + | Her2: 0 | Ki-67: <1% | - |
| B1 | 34 | Female | Adenosis of breast | - | - | - | Benign | - | - | ER: + + + | PR: + + + | Her2: 0 | Ki-67: 5% | - |
| B2 | 34 | Female | Adenosis of breast | - | - | - | Benign | - | - | ER: * | PR: * | Her2: * | Ki-67: * | - |
| B3 | 36 | Female | Adenosis of breast | - | - | - | Benign | - | - | ER: * | PR: * | Her2: * | Ki-67: * | - |
| B4 | 30 | Female | Adenosis of breast | - | - | - | Benign | - | - | ER: + + + | PR: + + + | Her2: 0 | Ki-67: 3% | - |
| B5 | 28 | Female | Adenosis of breast | - | - | - | Benign | - | - | ER: + + + | PR: + + + | Her2: 0 | Ki-67: 2% | - |
| B6 | 33 | Female | Adenosis of breast | - | - | - | Benign | - | - | ER: + + + | PR: + + + | Her2: 2 + | Ki-67: 7% | - |
| B7 | 23 | Female | Adenosis of breast | - | - | - | Benign | - | - | ER: + + + | PR: + + + | Her2: 3 + | Ki-67: 2% | - |
| B8 | 47 | Female | Adenosis of breast | - | - | - | Benign | - | - | ER: + + + | PR: + + + | Her2: 0 | Ki-67: 5% | - |
| B9 | 27 | Female | Breast fibroadenoma | - | - | - | Benign | - | - | ER: + + + | PR: + + + | Her2: 2 + | Ki-67: 3% | - |
| B10 | 42 | Female | Breast fibroadenoma | - | - | - | Benign | - | - | ER: + + + | PR: + + + | Her2: 0 | Ki-67: <1% | - |

| B11 | 21 | Female | Breast fibroadenoma | - | - | - | Benign | - | - | ER: + + + | PR: + + + | Her2: 0 | Ki-67: 7% | - |
| --- | --- | --- | --- | --- | --- | --- | --- | --- | --- | --- | --- | --- | --- | --- |
| B12 | 38 | Female | Breast fibroadenoma | - | - | - | Benign | - | - | ER: + + + | PR: + + + | Her2: 1 + | Ki-67: <1% | - |
| B13 | 19 | Female | Breast fibroadenoma | - | - | - | Benign | - | - | ER: + + + | PR: - | Her2: 0 | Ki-67: 7% | - |
| B14 | 48 | Female | DCIS | - | TisN0M0 | 0 | Cancer | 3*2*1.5 | Negative | ER: - | PR: - | Her2: 3 + | Ki-67: 9% | Her-2 type |
| B15 | 48 | Female | DCIS | - | TisN0M0 | 0 | Cancer | - | Negative | ER: - | PR: - | Her2: 3 + | Ki-67: <1% | Her-2 type |
| C1 | 38 | Female | Invasive lo bular  carcinoma | - | T3N1M0 | IIIA | Cancer | 2*2*1 | Positive | ER: + + | PR: + | Her2: 3 + | Ki-67: 8% | Luminal B |
| C2 | 51 | Female | Invasive lo bular  carcinoma | - | T2N1M0 | IIB | Cancer | 3*3 | Positive | ER: - | PR: + + | Her2: 2 + | Ki-67: 7% | Luminal A |
| C3 | 58 | Female | Invasive lo bular  carcinoma | - | T3N1M0 | IIIA | Cancer | - | Positive | ER: + + | PR: - | Her2: 0 | Ki-67: <1% | Luminal A |
| C4 | 61 | Female | Invasive lo bular  carcinoma | - | T2N2M0 | IIIA | Cancer | 3.3*3*2.7 | Positive | ER: + + + | PR: + + + | Her2: 1 + | Ki-67: * | Luminal A |
| C5 | 40 | Female | Invasive lo bular  carcinoma | - | T3N3M0 | IIIC | Cancer | 6*5*3 | Positive | ER: + + + | PR: + + + | Her2: 0 | Ki-67: 15% | Luminal A |
| C6 | 62 | Female | Non-specific invasive breast cancer | 1--2 | T2N2M0 | IIIA | Cancer | 4*3.5*3 | Positive | ER: + + + | PR: - | Her2: 0 | Ki-67: 65% | Luminal A |
| C7 | 69 | Female | Non-specific invasive breast cancer | 1 | T2N2M0 | IIIA | Cancer | 2.5*2*1.5 | Positive | ER: + + + | PR: - | Her2: 2 + | Ki-67: 8% | Luminal A |
| C8 | 37 | Female | Non-specific invasive breast cancer | 1 | T2N2M0 | IIIA | Cancer | 3.5*3*3 | Positive | ER: + + | PR: - | Her2: 3 + | Ki-67: <1% | Luminal B |

| C9 | 50 | Female | Non-specific invasive breast cancer | 1 | T2N1M0 | IIB | Cancer | 2.5*2.5*1.5 | Positive | ER: + + + | PR: + + + | Her2: 1 + | Ki-67: 20% | Luminal A |
| --- | --- | --- | --- | --- | --- | --- | --- | --- | --- | --- | --- | --- | --- | --- |
| C10 | 40 | Female | Non-specific invasive breast cancer | 1--2 | T2N0M0 | IIA | Cancer | 2*2*1.5 | Negative | ER: + + | PR: + + | Her2: 1 + | Ki-67: 3% | Luminal A |
| C11 | 25 | Female | Non-specific invasive breast cancer | 1--2 | T2N2M0 | IIIA | Cancer | 4.5*3.5*3 | Positive | ER: + + + | PR: + + + | Her2: 0 | Ki-67: 5% | Luminal A |
| C12 | 55 | Female | Non-specific invasive breast cancer | 1--2 | T2N2M0 | IIIA | Cancer | 3.9*3.6*3 | Positive | ER: + + + | PR: + + + | Her2: 0 | Ki-67: 15% | Luminal A |
| C13 | 51 | Female | Non-specific invasive breast cancer | 1--2 | T2N1M0 | IIB | Cancer | 4*3*2.5 | Positive | ER: - | PR: - | Her2: 3 + | Ki-67: <1% | Her-2 type |
| C14 | 27 | Female | Non-specific invasive breast cancer | 2 | T2N1M0 | IIB | Cancer | 3.4*3.3*3 | Positive | ER: - | PR: - | Her2: 3 + | Ki-67: 2% | Her-2 type |
| C15 | 58 | Male | Non-specific invasive breast cancer | 2 | T2N2M0 | IIIA | Cancer | 3.5*3.5*3.2 | Positive | ER: + + + | PR: + | Her2: 0 | Ki-67: <1% | Luminal A |
| D1 | 54 | Female | Non-specific invasive breast cancer | 2 | T2N0M0 | IIA | Cancer | 2.7*2.*2 | Negative | ER: + + + | PR: - | Her2: 3 + | Ki-67: 35% | Luminal B |
| D2 | 71 | Female | Non-specific invasive breast cancer | 2 | T2N1MO | IIB | Cancer | - | Positive | ER: - | PR: - | Her2: 0 | Ki-67: 10% | TNBC |
| D3 | 60 | Female | Non-specific invasive breast cancer | 2 | T2N0M0 | IIA | Cancer | - | Negative | ER: - | PR: - | Her2: 3 + | Ki-67: 1% | Her-2 type |
| D4 | 55 | Female | Non-specific invasive breast cancer | 2 | T2N1M0 | IIB | Cancer | 4.5*3*2 | Positive | ER: + + + | PR: + + + | Her2: 3 + | Ki-67: 3% | Luminal B |
| D5 | 73 | Female | Non-specific invasive breast cancer | 2 | T2N2M0 | IIIA | Cancer | 3.5*3*1.8 | Positive | ER: + + + | PR: - | Her2: 1 + | Ki-67: 20% | Luminal A |
| D6 | 62 | Female | Non-specific invasive breast cancer | 2 | T2N0M0 | IIA | Cancer | 3*3*2.5 | Negative | ER: - | PR: - | Her2: 3 + | Ki-67: 15% | Her-2 type |

| D7 | 62 | Male | Non-specific invasive breast cancer | 2 | T4aN0M0 | IIIB | Cancer | 5.8*3 | Negative | ER: + + + | PR: + + + | Her2: 1 + | Ki-67: 4% | Luminal A |
| --- | --- | --- | --- | --- | --- | --- | --- | --- | --- | --- | --- | --- | --- | --- |
| D8 | 57 | Female | Non-specific invasive breast cancer | 2 | T2N2M0 | IIIA | Cancer | 2.5*2*2 | Positive | ER: + | PR: - | Her2: 3 + | Ki-67: 10% | Luminal B |
| D9 | 49 | Female | Non-specific invasive breast cancer | 2 | T2N2M0 | IIIA | Cancer | 3*3*3 | Positive | ER: - | PR: - | Her2: 3 + | Ki-67: 40% | Her-2 type |
| D10 | 63 | Female | Non-specific invasive breast cancer | 2 | T2N2M0 | IIIA | Cancer | 3.7*3.2*3 | Positive | ER: + + + | PR: + + | Her2: 1 + | Ki-67: 15% | Luminal A |
| D11 | 35 | Female | Non-specific invasive breast cancer | 2 | T4N2M0 | IIIB | Cancer | - | Positive | ER: + + + | PR: + + + | Her2: 1 + | Ki-67: 25% | Luminal A |
| D12 | 49 | Female | Non-specific invasive breast cancer | 2 | T3N1M0 | IIIA | Cancer | 3*2.5*2 | Positive | ER: - | PR: + + | Her2: 3 + | Ki-67: 35% | Luminal B |
| D13 | 50 | Female | Non-specific invasive breast cancer | 2 | T2N2M0 | IIIA | Cancer | 4.5*4*2 | Positive | ER: + + | PR: - | Her2: 3 + | Ki-67: 6% | Luminal B |
| D14 | 57 | Female | Non-specific invasive breast cancer | 2 | T1cN2M0 | IIIA | Cancer | 2*1.5*1.5 | Positive | ER: - | PR: - | Her2: 1 + | Ki-67: 30% | TNBC |
| D15 | 47 | Female | Non-specific invasive breast cancer | 2 | T2N2M0 | IIIA | Cancer | 4*4.5*1 | Positive | ER: + + + | PR: - | Her2: 3 + | Ki-67: 2% | Luminal B |
| E1 | 64 | Female | Non-specific invasive breast cancer | 2 | T2N1M0 | IIB | Cancer | 4*3.5*3.5 | Positive | ER: - | PR: - | Her2: 3 + | Ki-67: 50% | Her-2 type |
| E2 | 43 | Female | Non-specific invasive breast cancer | 2 | T2N3M0 | IIIC | Cancer | 5*3*2 | Positive | ER: - | PR: - | Her2: 3 + | Ki-67: 65% | Her-2 type |
| E3 | 51 | Female | Non-specific invasive breast cancer | 2 | T2N2M0 | IIIA | Cancer | 3.5*3*2.5 | Positive | ER: + + + | PR: + + + | Her2: 3 + | Ki-67: 40% | Luminal B |
| E4 | 55 | Female | Non-specific invasive breast cancer | 2 | T2N3M0 | IIIC | Cancer | - | Positive | ER: + | PR: - | Her2: 2 + | Ki-67: 0.7 | Luminal B |

| E5 | 39 | Female | Non-specific invasive breast cancer | 2 | T4N3M0 | IIIC | Cancer | 5*3*2 | Positive | ER: + + + | PR: + + + | Her2: 2 + | Ki-67: 30% | Luminal B |
| --- | --- | --- | --- | --- | --- | --- | --- | --- | --- | --- | --- | --- | --- | --- |
| E6 | 38 | Female | Non-specific invasive breast cancer | 2 | T2N1M0 | IIB | Cancer | - | Positive | ER: - | PR: - | Her2: 3 + | Ki-67: 20% | Her-2 type |
| E7 | 56 | Female | Non-specific invasive breast cancer | 2 | T2N3M0 | IIIC | Cancer | 3.5*2.5*2 | Positive | ER: - | PR: - | Her2: 3 + | Ki-67: 10% | Her-2 type |
| E8 | 61 | Female | Non-specific invasive breast cancer | 2 | T2N3M0 | IIIC | Cancer | 5.5*3*2.5 | Positive | ER: - | PR: - | Her2: 2 + | Ki-67: 65% | Her-2 type |
| E9 | 64 | Female | Non-specific invasive breast cancer | 2 | T1N2M0 | IIIA | Cancer | 1.5*1.5*1.5 | Positive | ER: + + + | PR: + + + | Her2: 0 | Ki-67: <1% | Luminal A |
| E10 | 43 | Female | Non-specific invasive breast cancer | 2 | T2N1M0 | IIB | Cancer | 3*2.5*2 | Positive | ER: - | PR: - | Her2: 3 + | Ki-67: <1% | Her-2 type |
| E11 | 53 | Female | Non-specific invasive breast cancer | 2 | T3N3M0 | IIIC | Cancer | 7*7*4 | Positive | ER: - | PR: - | Her2: 3 + | Ki-67: 10% | Her-2 type |
| E12 | 48 | Female | Non-specific invasive breast cancer | 2 | T2N0M0 | IIA | Cancer | 3*2.8*2.5 | Negative | ER: + + + | PR: - | Her2: 3 + | Ki-67: 20% | Luminal B |
| E13 | 58 | Female | Non-specific invasive breast cancer | 2 | T2N2M0 | IIIA | Cancer | 3.9*3*3 | Positive | ER: + + + | PR: - | Her2: 3 + | Ki-67: <1% | Luminal B |
| E14 | 52 | Female | Non-specific invasive breast cancer | 2 | T2N0M0 | IIA | Cancer | 3*2.8*2 | Negative | ER: + + + | PR: + | Her2: 2 + | Ki-67: 45% | Luminal B |
| E15 | 66 | Female | Non-specific invasive breast cancer | 2 | T2N0M0 | IIA | Cancer | 3.5*3*3 | Negative | ER: + + + | PR: - | Her2: 3 + | Ki-67: <1% | Luminal B |
| F1 | 53 | Female | Non-specific invasive breast cancer | 2 | T2N1M0 | IIB | Cancer | 3.5*3.5*3 | Positive | ER: + + + | PR: + + + | Her2: 0 | Ki-67: 10%8 | Luminal A |
| F2 | 57 | Female | Non-specific invasive breast cancer | 2 | T3N1M0 | IIIA | Cancer | - | Positive | ER: - | PR: - | Her2: 3 + | Ki-67: 35% | Her-2 type |

| F3 | 43 | Female | Non-specific invasive breast cancer | 2 | T2N2M0 | IIIA | Cancer | 3.8*3.2*3.2 | Positive | ER: - | PR: - | Her2: 0 | Ki-67: 10% | TNBC |
| --- | --- | --- | --- | --- | --- | --- | --- | --- | --- | --- | --- | --- | --- | --- |
| F4 | 48 | Female | Non-specific invasive breast cancer | 2 | T2N1M0 | IIB | Cancer | 4*3.8*3.5 | Positive | ER: + + + | PR: + + + | Her2: 0 | Ki-67: <1% | Luminal A |
| F5 | 54 | Female | Non-specific invasive breast cancer | 2 | T2N1M0 | IIB | Cancer | 3.6*3*2.5 | Positive | ER: - | PR: - | Her2: 2 + | Ki-67: <1% | Her-2 type |
| F6 | 41 | Female | Non-specific invasive breast cancer | 2 | T4N2M0 | IIIB | Cancer | 3*2.5*2.5 | Positive | ER: + + + | PR: + + | Her2: 0 | Ki-67: <1% | Luminal A |
| F7 | 56 | Female | Non-specific invasive breast cancer | 2 | T2N2M0 | IIIA | Cancer | 4.5*3*2.8 | Positive | ER: + + | PR: + + + | Her2: 0 | Ki-67: <1% | Luminal A |
| F8 | 54 | Female | Non-specific invasive breast cancer | 2 | T2N1M0 | IIB | Cancer | 4*3.5*3 | Positive | ER: - | PR: - | Her2: 2 + | Ki-67: <1% | Her-2 type |
| F9 | 46 | Female | Non-specific invasive breast cancer | 2 | T2N1M0 | IIB | Cancer | 3.8*3.3*3 | Positive | ER: + | PR: + + | Her2: 0 | Ki-67: 2% | Luminal A |
| F10 | 28 | Female | Non-specific invasive breast cancer | 2 | T2N1M0 | IIB | Cancer | 3.7*3.2*3 | Positive | ER: + | PR: + + + | Her2: 3 + | Ki-67: <1% | Luminal B |
| F11 | 44 | Female | Non-specific invasive breast cancer | 2 | T2N1M0 | IIB | Cancer | 4.5*3.8*3.5 | Positive | ER: - | PR: - | Her2: 0 | Ki-67: 20%5 | TNBC |
| F12 | 50 | Female | Non-specific invasive breast cancer | 2 | T2N1M0 | IIB | Cancer | 3.8*3*3 | Positive | ER: - | PR: - | Her2: 0 | Ki-67: <1% | TNBC |
| F13 | 40 | Female | Non-specific invasive breast cancer | 2 | T2N1M0 | IIB | Cancer | 3.9*3.5*3.2 | Positive | ER: - | PR: - | Her2: 3 + | Ki-67: 20% | Her-2 type |
| F14 | 54 | Female | Non-specific invasive breast cancer | 2 | T2N1M0 | IIB | Cancer | 3.9*3.6*3.3 | Positive | ER: - | PR: - | Her2: 3 + | Ki-67: 20% | Her-2 type |
| F15 | 57 | Female | Non-specific invasive breast cancer | 2 | T2N2M0 | IIIA | Cancer | 3*2*2 | Positive | ER: + + + | PR: - | Her2: 1 + | Ki-67: <1% | Luminal A |

| G1 | 50 | Female | Non-specific invasive breast cancer | 2 | T2N2M0 | IIIA | Cancer | 5*5*3 | Positive | ER: - | PR: - | Her2: 3 + | Ki-67: 15% | Her-2 type |
| --- | --- | --- | --- | --- | --- | --- | --- | --- | --- | --- | --- | --- | --- | --- |
| G2 | 52 | Female | Non-specific invasive breast cancer | 2 | T2N3M0 | IIIC | Cancer | 2.5*2.5*2 | Positive | ER: + + + | PR: + + | Her2: 2 + | Ki-67: 75% | Luminal B |
| G3 | 47 | Female | Non-specific invasive breast cancer | 2 | T2N0M0 | IIA | Cancer | 2.5*2*1.5 | Negative | ER: + + + | PR: + + + | Her2: 3 + | Ki-67: 8% | Luminal B |
| G4 | 41 | Female | Non-specific invasive breast cancer | 2 | T3N3M0 | IIIC | Cancer | 8*6*3 | Positive | ER: - | PR: - | Her2: 3 + | Ki-67: 20% | Her-2 type |
| G5 | 65 | Female | Non-specific invasive breast cancer | 2 | T2N0M0 | IIA | Cancer | 3*2.5*1.5 | Negative | ER: + + + | PR: + + + | Her2: 1 + | Ki-67: 15% | Luminal A |
| G6 | 45 | Female | Non-specific invasive breast cancer | 2 | T2N2M0 | IIIA | Cancer | 4*4*3 | Positive | ER: - | PR: - | Her2: 3 + | Ki-67: 20% | Her-2 type |
| G7 | 14 | Female | Non-specific invasive breast cancer | 2 | T2N1M0 | IIB | Cancer | 3*3*2.5 | Positive | ER: + + + | PR: + + + | Her2: 2 + | Ki-67: 15% | Luminal B |
| G8 | 31 | Female | Non-specific invasive breast cancer | 2 | T2N0M0 | IIA | Cancer | 4.5*4*2.5 | Negative | ER: + + + | PR: + + + | Her2: 3 + | Ki-67: 35% | Luminal B |
| G9 | 50 | Female | Non-specific invasive breast cancer | 2 | T2N2M0 | IIIA | Cancer | 5*4.5*3.5 | Positive | ER: - | PR: - | Her2: 3 + | Ki-67: 35% | Her-2 type |
| G10 | 47 | Female | Non-specific invasive breast cancer | 2 | T2N1M0 | IIB | Cancer | 4*3*3 | Positive | ER: - | PR: - | Her2: 0 | Ki-67: 20% | TNBC |
| G11 | 43 | Female | Non-specific invasive breast cancer | 2 | T2N2M0 | IIIA | Cancer | 3.7*3.5*3 | Positive | ER: + + + | PR: + + + | Her2: 0 | Ki-67: <1% | Luminal A |
| G12 | 35 | Female | Non-specific invasive breast cancer | 2 | T2N2M0 | IIIA | Cancer | 3*3*2.5 | Positive | ER: + + + | PR: + + + | Her2: 0 | Ki-67: 8% | Luminal A |
| G13 | 47 | Female | Non-specific invasive breast cancer | 2 | T2N2M0 | IIIA | Cancer | 3*2.5*2.5 | Positive | ER: + + + | PR: + + | Her2: 3 + | Ki-67: 35% | Luminal B |

| G14 | 50 | Female | Non-specific invasive breast cancer | 2 | T2N2M0 | IIIA | Cancer | 4.5*4*3 | Positive | ER: + + + | PR: + + + | Her2: 1 + | Ki-67: 20% | Luminal A |
| --- | --- | --- | --- | --- | --- | --- | --- | --- | --- | --- | --- | --- | --- | --- |
| G15 | 44 | Female | Non-specific invasive breast cancer | 2 | T2N1M0 | IIB | Cancer | 4.5*3.5*3 | Positive | ER: + | PR: - | Her2: 2 + | Ki-67: 40% | Luminal B |
| H1 | 56 | Female | Non-specific invasive breast cancer | 2 | T2N2M0 | IIIA | Cancer | 3.9*3.2*3 | Positive | ER: + + | PR: - | Her2: 3 + | Ki-67: 2% | Luminal B |
| H2 | 84 | Female | Non-specific invasive breast cancer | 2 | T2N2M0 | IIIA | Cancer | 3.4*3.2*3.2 | Positive | ER: + + + | PR: - | Her2: 0 | Ki-67: <1% | Luminal A |
| H3 | 54 | Female | Non-specific invasive breast cancer | 2 | T3N1M0 | IIIA | Cancer | - | Positive | ER: + + | PR: - | Her2: 3 + | Ki-67: 35% | Luminal B |
| H4 | 63 | Female | Non-specific invasive breast cancer | 2 | T2N1M0 | IIB | Cancer | 3.5*3*2 | Positive | ER: + + + | PR: + + + | Her2: 3 + | Ki-67: 45% | Luminal B |
| H5 | 88 | Female | Non-specific invasive breast cancer | 2 | T2N2M0 | IIIA | Cancer | 3*2.5*2 | Positive | ER: - | PR: - | Her2: 0 | Ki-67: 35% | TNBC |
| H6 | 56 | Female | Non-specific invasive breast cancer | 2 | T3N2M0 | IIIA | Cancer | 5*4*3 | Positive | ER: - | PR: - | Her2: - | Ki-67: <1% | TNBC |
| H7 | 45 | Female | Non-specific invasive breast cancer | 2 | T2N0M0 | IIA | Cancer | 3*2.5*1 | Negative | ER: + + | PR: + + + | Her2: 3 + | Ki-67: 50% | Luminal B |
| H8 | 53 | Female | Non-specific invasive breast cancer | 2 | T1N1MO | IIA | Cancer | 4.5*4*4 | Positive | ER: + + + | PR: + + + | Her2: 1 + | Ki-67: 10% | Luminal A |
| H9 | 47 | Female | Non-specific invasive breast cancer | 2 | T2N2M0 | IIIA | Cancer | 4*3*2 | Positive | ER: + + + | PR: + + + | Her2: 1 + | Ki-67: 10% | Luminal A |
| H10 | 61 | Female | Non-specific invasive breast cancer | 2 | T2N2M0 | IIIA | Cancer | 3.9*3.2*3 | Positive | ER: + + + | PR: + | Her2: 3 + | Ki-67: 15% | Luminal B |
| H11 | 34 | Female | Non-specific invasive breast cancer | 2--3 | T4N2M0 | IIIB | Cancer 2 | .5*2.3 ；3.5* | Positive | ER: - | PR: - | Her2: 3 + | Ki-67: 75% | Her-2 type |

| H12 | 85 | Female | Non-specific invasive breast cancer | 2--3 | T2N1M0 | IIB | Cancer | 3.5*3.5*3.2 | Positive | ER: - | PR: - | Her2: 2 + | Ki-67: 45% | Her-2 type |
| --- | --- | --- | --- | --- | --- | --- | --- | --- | --- | --- | --- | --- | --- | --- |
| H13 | 58 | Female | Non-specific invasive breast cancer | 2--3 | T2N2M0 | IIIA | Cancer | 3.2*3*2.6 | Positive | ER: + + + | PR: - | Her2: 1 + | Ki-67: 3% | Luminal A |
| H14 | 42 | Female | Non-specific invasive breast cancer | 2--3 | T2N0M0 | IIA | Cancer | 3*2.5*1.5 | Negative | ER: - | PR: - | Her2: 3 + | Ki-67: 65% | Her-2 type |
| H15 | 62 | Female | Non-specific invasive breast cancer | 3 | T2N2M0 | IIIA | Cancer | 3.8*3.4*3 | Positive | ER: - | PR: - | Her2: 1 + | Ki-67: 35% | TNBC |
| I1 | 45 | Female | Non-specific invasive breast cancer | 3 | T2N2M0 | IIIA | Cancer | 3.9*3.6*3.4 | Positive | ER: - | PR: - | Her2: 0 | Ki-67: <1% | TNBC |
| I2 | 53 | Female | Non-specific invasive breast cancer | 3 | T2N0M0 | IIA | Cancer | - | Negative | ER: + | PR: - | Her2: 3 + | Ki-67: 8% | Luminal B |
| I3 | 30 | Female | Non-specific invasive breast cancer | 3 | T2N2M0 | IIIA | Cancer | 5*4*3 | Positive | ER: - | PR: - | Her2: 1 + | Ki-67: 30% | TNBC |
| I4 | 43 | Female | Non-specific invasive breast cancer | 3 | T2N2M0 | IIIA | Cancer | 4.5*3.5*3 | Positive | ER: - | PR: - | Her2: 1 + | Ki-67: 35% | TNBC |
| I5 | 33 | Female | Non-specific invasive breast cancer | 3 | T2N1M0 | IIB | Cancer | 4.8*3.8*3 | Positive | ER: - | PR: - | Her2: 0 | Ki-67: 55% | TNBC |
| I6 | 46 | Female | Non-specific invasive breast cancer | 3 | T2N0M0 | IIIA | Cancer | 3*3*2.5 | Negative | ER: + + + | PR: + + + | Her2: 3 + | Ki-67: 25% | Luminal B |
| I7 | 56 | Female | Non-specific invasive breast cancer | 3 | T2N0M0 | IIA | Cancer | 4*2.5*2 | Negative | ER: - | PR: - | Her2: 1 + | Ki-67: 50% | TNBC |
| I8 | 51 | Female | Non-specific invasive breast cancer | 3 | T2N1M0 | IIB | Cancer | 3.5*3.5*3 | Positive | ER: + + | PR: - | Her2: 1 + | Ki-67: 15% | Luminal A |
| I9 | 29 | Female | Non-specific invasive breast cancer | 3 | T2N1M0 | IIB | Cancer | 4*2.5*2 | Positive | ER: - | PR: - | Her2: 0 | Ki-67: 0.85 | TNBC |

| I10 | 32 | Female | Carcinoma with apocrine differentiation | - | T4N0M0 | IIIB | Cancer | - | Negative | ER: - | PR: - | Her2: 3 + | Ki-67: 30% | Her-2 type |
| --- | --- | --- | --- | --- | --- | --- | --- | --- | --- | --- | --- | --- | --- | --- |
| I11 | 20 | Female | Carcinoma with apocrine differentiation | - | T2N0M0 | IIA | Cancer | - | Negative | ER: - | PR: - | Her2: 3 + | Ki-67: 2% | Her-2 type |
| I12 | 53 | Female | Carcinoma with apocrine differentiation | - | T2N0M0 | IIA | Cancer | - | Negative | ER: - | PR: + | Her2: 0 | Ki-67: 4% | Luminal A |
| I13 | 57 | Female | Micropapillary carcinoma | - | T2N2M0 | IIIA | Cancer | 4.5*3*1.5 | Positive | ER: + + + | PR: - | Her2: 1 + | Ki-67: <1% | Luminal A |
| I14 | 42 | Female | Micropapillary carcinoma | - | T2N0M0 | IIA | Cancer | - | Negative | ER: + + | PR: - | Her2: 0 | Ki-67: 0.7 | Luminal A |
| I15 | 46 | Female | Cribriform carcinoma | - | T2N0M0 | IIA | Cancer | 2.5*2.5*2 | Negative | ER: + + + | PR: - | Her2: 0 | Ki-67: 10% | Luminal A |
| J1 | 47 | Female | Cribriform carcinoma | - | T2N0M0 | IIA | Cancer | 3*2*1.5 | Negative | ER: - | PR: - | Her2: 3 + | Ki-67: 7% | Her-2 type |
| J2 | 53 | Female | Mucinous carcinoma | - | T3N1M0 | IIIA | Cancer | - | Positive | ER: + + | PR: - | Her2: 0 | Ki-67: 8% | Luminal A |
| J3 | 58 | Female | Mucinous carcinoma | - | T2N1M0 | IIB | Cancer | - | Positive | ER: + + + | PR: + + + | Her2: 3 + | Ki-67: 30% | Luminal B |
| J4 | 82 | Female | Metaplastic carcinoma | - | T3N2M0 | IIIA | Cancer | - | Positive | ER: - | PR: - | Her2: 0 | Ki-67: 30% | TNBC |
| J5 | 54 | Female | Metaplastic carcinoma | - | T2N0M0 | IIA | Cancer | 3*2*1.5 | Negative | ER: - | PR: - | Her2: 1 + | Ki-67: 35% | TNBC |
| J6 | 56 | Female | Carcinoma with medullary features | - | T2N2M0 | IIIA | Cancer | 4.5*3.5*3 | Positive | ER: - | PR: - | Her2: 0 | Ki-67: 20% | TNBC |
| J7 | 52 | Female | Carcinoma with medullary features | - | T2N0M0 | IIA | Cancer | - | Negative | ER: - | PR: - | Her2: 3 + | Ki-67: 15% | Her-2 type |

| J8 | 46 | Female | Carcinoma with medullary features | - | T3N0M0 | IIB | Cancer | - | Negative | ER: - | PR: - | Her2: 3 + | Ki-67: 50% | Her-2 type |
| --- | --- | --- | --- | --- | --- | --- | --- | --- | --- | --- | --- | --- | --- | --- |
| J9 | 60 | Female | Carcinoma with medullary features | - | T4BN1M0 | IIIB | Cancer | - | Positive | ER: - | PR: - | Her2: 3 + | Ki-67: <1% | Her-2 type |
| J10 | 57 | Female | Carcinoma with medullary features | - | T2N0M0 | IIA | Cancer | 4*4*4 | Negative | ER: - | PR: - | Her2: 3 + | Ki-67: 20%5 | Her-2 type |
| J11 | 56 | Female | Carcinoma with medullary features | - | T2N0M0 | IIA | Cancer | - | Negative | ER: - | PR: - | Her2: 3 + | Ki-67: 10% | Her-2 type |
| J12 | 43 | Female | Carcinoma with medullary features | - | T1N0M0 | IA | Cancer | 2*2*2 | Negative | ER: + + + | PR: + + | Her2: 3 + | Ki-67: 30% | Luminal B |
| J13 | 63 | Female | Carcinoma with medullary features | - | T2N1M0 | IIB | Cancer | 3*3*2.5 | Positive | ER: - | PR: - | Her2: 0 | Ki-67: 40% | TNBC |
| J14 | 69 | Female | Carcinoma with  neuroendocrine  characteristics | - | T2N1M0 | IIB | Cancer | - | Positive | ER: - | PR: - | Her2: 0 | Ki-67: 10% | TNBC |
| J15 | 65 | Female | Carcinoma with  neuroendocrine  characteristics | - | T2N1M0 | IIB | Cancer | - | Positive | ER: - | PR: - | Her2: 3 + | Ki-67: 30% | Her-2 type |
| J16 | 69 | Female | Carcinoma with  neuroendocrine  characteristics | - | T2N1M0 | IIB | Cancer | - | Positive | ER: - | PR: - | Her2: 0 | Ki-67: <1% | TNBC |

**STable 2.** List of BRAC tissues with molecular classification according to National Comprehensive Cancer Network guideline in 2021.
