# Supplementary material for: 25HC regulates the polarization of CD163+ macrophages in the immune microenvironment of triple-negative breast cancer through the interferon pathway
Source: Front Immunol. 2026 May 28;17:1812056. doi: 10.3389/fimmu.2026.1812056 (PMC13253280; doi:10.3389/fimmu.2026.1812056)
Supplement: Supplementary file 5 [file Table3.docx]

| ***Gene Name*** | **Primer sequence** |
| --- | --- |
| *CH25H* | 5’-3’CCCTTGGTCCACTCACAGAC,3’-5’ACGGAGCGAAGTTGCAGTTA |
| *IL-1β* | 5’-3’TGAGCTCGCCAGTGAAATGA,3’-5’AGATTCGTAGCTGGATGCCG |
| *IL-6* | 5’-3’CCACCGGGAACGAAAGAGAA,3’-5’GAGAAGGCAACTGGACCGAA |
| *IL-10* | 5’-3’AGGGCACCCAGTCTGAGAAC,3’-5’TTCACTCTGCTGAAGGCATCT |
| *IRF7* | 5’-3 ’ AGCCCTTACCTCCCCTGTTA,3’-5’TGCCCTCTCAGGAGCCAA |
| *GAPDH* | 5’-3 ’ GACAGTCAGCCGCATCTTCT,3’-5’GCGCCCAATACGACCAAATC |
| *TNF-α* | 5’-3’CACAGTGAAGTGCTGGCAAC,3’-5’GATCAAAGCTGTAGGCCCCA |
| *NOS2* | 5’-3’CGCATGACCTTGGTGTTTGG,3’-5’CATAGACCTTGGGCTTGCCA |
| *CSF1R* | 5’-3’TGCGGCCAGGCTAAAAGGG,3’-5’GGCTGTTTGTCTTGTTTTCCTCTT |
| *CD68* | 5’-3’ACAGGGAATGACTGTCCTCAC,3’-5’CAGTGCTCTCTGTAACCGTGG |
| *CD11b* | 5’-3’GCTTTGGTGGCTTCCTTGTG,3’-5’CTGGAAGGAGCCAGAACCTG |
| *CD11c* | 5’-3’TGGCTTCTTCAAGCGTCAGT,3’-5’GAGGGTAATGGGGAGTGGGC |
| *CD80* | 5’-3’TCAGAAGTGGAGTCTTACCCTG,3’-5’CCTGGGTCTCCAAAGGTTGT |
| *CD86* | 5’-3’GGACTAGCACAGACACACGGA,3’-5’CTTCAGAGGAGCAGCACCAGA |
| *CD163* | 5’-3’GAGACAGCGGCTTGCAGTTT,3’-5’CAGCTGACTCATGGGAATTTTCT |
| *CD206* | 5’-3’CCAAACGCCTTCATTTGCCA,3’-5’ACCTTCCTTGCACCCTGATG |
| *CD14* | 5’-3’AAGCACTTCCAGAGCCTGTC,3’-5’TCGTCCAGCTCACAAGGTTC |

**STable 1.** List of PCR primers used in this study.
